# Tubule-mitophagic secretion of SerpinG1 reprograms macrophages to instruct anti-septic acute kidney injury efficacy of high-dose ascorbate mediated by NRF2 transactivation

**DOI:** 10.7150/ijbs.74430

**Published:** 2022-08-08

**Authors:** Yin Ni, Guo-Hua Wu, Juan-Juan Cai, Run Zhang, Yang Zheng, Jing-Quan Liu, Xiang-Hong Yang, Xue Yang, Ye Shen, Jun-Mei Lai, Xiang-Ming Ye, Shi-Jing Mo

**Affiliations:** 1 Emergency and Intensive Care Unit Center, Department of Intensive Care Unit, Zhejiang Provincial People's Hospital, Affiliated People's Hospital, Hangzhou Medical College, Hangzhou 310014, Zhejiang, P.R.China.; 2 Zhejiang University School of Medicine, Zhejiang University, Hangzhou 310029, Zhejiang, P.R.China.; 3 Department of Pathology, Zhejiang Provincial People's Hospital, Affiliated People's Hospital, Hangzhou Medical College, Hangzhou 310014, Zhejiang, P.R.China.; 4 Clinical Research Institute, Zhejiang Provincial People's Hospital, Affiliated People's Hospital, Hangzhou Medical College, Hangzhou 310014, Zhejiang, P.R.China.; 5 Center for Rehabilitation Medicine, Department of Intensive Rehabilitation Care Unit, Zhejiang P rovincial People's Hospital, Affiliated People's Hospital, Hangzhou Medical College, Hangzhou 310014, Zhejiang, P.R.China.; 6 Center for Rehabilitation Medicine, Rehabilitation & Sports Medicine Research Institute of Zhejiang Province, Department of Rehabilitation Medicine, Zhejiang Provincial People's Hospital, Affiliated People's Hospital, Hangzhou Medical College, Hangzhou 310014, Zhejiang, P.R.China.

**Keywords:** septic acute kidney injury, tubular mitophagy, macrophage, high-dose ascorbate, serpin family G member 1

## Abstract

High-dose ascorbate confers tubular mitophagy responsible for septic acute kidney injury (AKI) amelioration, yet its biological roles in immune regulation remain poorly understood.

**Methods:** The role of tubular mitophagy in macrophage polarization upon high-dose ascorbate treatment was assessed by fluorescence-activated cell sorter analysis (FACS) *in vitro* and by immunofluorescence in AKI models of LPS-induced endotoxemia (LIE) from *Pax8-cre*; *Atg7*^flox/flox^ mice. The underlying mechanisms were revealed by RNA-sequencing, gene set enrichment analysis (GSEA), luciferase reporter, chromatin immunoprecipitation (ChIP) and adeno-associated viral vector serotype 9 (AAV9) delivery assays.

**Results:** High-dose ascorbate enables conversion of macrophages from a pro-inflammatory M1 subtype to an anti-inflammatory M2 subtype in murine AKI models of LIE, leading to decreased renal IL-1β and IL-18 production, reduced mortality and alleviated tubulotoxicity. Blockade of tubular mitophagy abrogates anti-inflammatory macrophages polarization under the high-dose ascorbate-exposed coculture systems. Similar abrogations are verified in LIE mice with tubular epithelium-specific ablation of *Atg7,* where the high-dose ascorbate-inducible renal protection and survival improvement are substantially weaker than their control littermates. Mechanistically, high-dose ascorbate stimulates tubular secretion of serpin family G member 1 (SerpinG1) through maintenance of mitophagy, for which nuclear factor-erythroid 2 related factor 2 (NRF2) transactivation is required. SerpinG1 perpetuates anti-inflammatory macrophages to prevent septic AKI, while kidney-specific disruption of SerpinG1 by adeno-associated viral vector serotype 9 (AAV9)-short hairpin RNA (shRNA) delivery thwarts the anti-inflammatory macrophages polarization and anti-septic AKI efficacy of high-dose ascorbate.

**Conclusion:** Our study identifies SerpinG1 as an intermediate of tubular mitophagy-orchestrated myeloid function during septic AKI and reveals a novel rationale for ascorbate-based therapy.

## Introduction

Acute kidney injury (AKI) represents the devastating complication of sepsis in intensive care unit (ICU) and intensive rehabilitation care unit (IRCU), with progressive systemic inflammatory response syndrome (SIRS), deleterious shock, severe renal failure and high mortality being implicated as the common features 1. Loss of kidney function resulting from sepsis-initiated tubular damage is an extremely complex pathophysiological process that involves host's innate and adaptive immune response to infection along with activation of residential and infiltrated leukocytes 2. Better understanding of the detailed mechanisms whereby tubular epithelial cells crosstalk with inflammatory leukocytes would be helpful for developing innovative strategy to overcome this destructive disease.

Infiltrating macrophages are prominent components of inflammatory microenvironment (IME) that affect various aspects of sepsis 3. Mounting literature document that elimination of macrophages during the early phase of infection delays injury repair, while adoptive transfer of macrophages potentiates antibacterial defense against sepsis 4-7. IME signals the pro- and anti-inflammatory polarization of macrophages that implement the cytotoxic or cytoprotective functions through expression of distinct proteins.

Pharmacological dosing of ascorbate has been emerged as a safe, low-toxic and tolerable therapeutic regimen for sepsis in randomized, double-blind trials of human subjects and preclinical studies of mice 8-12. Poor ascorbate status is associated with severity and metabolic dysfunction of endotoxemia, which blocks ascorbate absorption via gut inflammation 13, 14. In mammalian cells, ascorbate is transported across cellular membrane by sodium vitamin C cotransporter-1 and -2 (SVCT-1 and -2) encoding by *Slc23a1* and* Slc23a2* gene, respectively 15. In addition to function as a scavenger of reactive oxygen species (ROS), ascorbate inhibits cell apoptosis and sustains endothelial integrity 16. Our recent study highlight that high-dose ascorbate renders the PTEN-induced kinase 1/parkin RBR E3 ubiquitin protein ligase (PINK1/PARK2)-mediated tubular mitophagy to protect endotoxemic mice against septic AKI 17. Despite extensive investigations, however, the detailed mechanisms of how ascorbate influences renal IME is largely unknown, and the intrinsic linkage between tubular mitophagy and immunomodulatory functions of high-dose ascorbate during septic AKI has not been explored. In this study, we examine the regulatory role of tubular mitophagy in renal IME of LIE mice receiving high-dose ascorbate therapy and discover that high-dose ascorbate-inducible SerpinG1 perpetuates the anti-inflammatory polarization of macrophages in a tubular mitophagy-dependent fashion through nuclear factor-erythroid 2 related factor 2 (NRF2) transactivation. This tubular mitophagy-macrophages interplay thus provides an immunotherapeutic target specifically for the anti-septic AKI efficacy of high-dose ascorbate.

## Materials and Methods

### Mice and *in vivo* procedures

Transgenic B6.129P2(Cg)-*Pax8^tm1.1(cre)Mbu^*/J mice (Stock No. 028196) expressing the Cre recombinase under the control of a modified *Pax8* promoter that directs Cre expression predominantly in kidney tubular epithelium were purchased from Jackson Laboratory^18^. The *Atg7*^flox/flox^ mouse strain was purchased from the RIKEN BioResource Center (BRC No. RBRC02759) 19. The homozygous* Atg7*^ΔTE^ mice were generated by cross-breeding *Atg7*^flox/flox^ mice with B6.129P2(Cg)-*Pax8^tm1.1(cre)Mbu^*/J mice and backcrossing. The LPS-induced endotoxemia (LIE) models were established by a single intraperitoneal (i.p.) injection (20 mg/kg) of *Escherichia coli* 0111:B4 LPS (LPS) into mice as described previously 17, 20. All mice were received s.c. injection of imipenem/cilastatin (20 mg/kg) at the time of LIE challenge.

For macrophage elimination, mice were anesthetized by ketamine (100 mg/kg) and were suspended on a flat board and placed in a prone position with the ventral surface and rostrum facing downward. Mice were initiated injected with a suspension of 200 μL commercially available clodronate liposomes via tail-vein and then received the secondary injection 24 h later, followed by i.p. administration of PBS, low-dose ascorbate (0.6 g/kg) and high-dose ascorbate (6 g/kg), respectively. The efficiency of macrophage elimination was evaluated by sorting cells labeled with F4/80 antibody via flow cytometry. To reconstitute macrophages, macrophages (1×10^6^) isolated from the PBS- or high-dose ascorbate-treated mice were adoptively transferred by injection (i.v.) into the clodronate liposomes-pretreated mice, which were then subjected to LIE at three hours after macrophage reconstitution. Detailed schemes were indicated in the respective figures.

To examine the role of tubular mitophagy in regulating macrophages polarization and anti-septic AKI efficacy of high-dose ascorbate, the *Atg7*^flox/flox^ and *Atg7*^ΔTE^ mice were injected i.p. with ascorbate (6 g/kg) before being subjected to LIE. The high-dose ascorbate (b.i.d) was then given to mice for 72 h.

To evaluate the therapeutic value of SerpinG1, after 4 h of i.p. injection of LPS, mice were randomized to repetitive administration (q.d) of vehicle mixture (10 mg/mL glycine, 2.9 mg/mL sodium citrate and 8.5 mg/mL sodium chloride, pH 7.0) or 800 μg recombinant SerpinG1 (OPCA00220, Aviva Systems Biology) by tail-vein for 72 h. To explore the role of macrophages in anti-septic AKI efficacy of rSerpinG1, the clodronate liposomes-pretreated mice were subjected to LIE challenge for 4 h and then received 800 μg rSerpinG1 treatment.

For AAV9 delivery, AAV9 vector plasmids harboring either *SerpinG1* shRNA or the scrambled shRNA were administered to mice at 1 × 10^12^ copies via renal vein, which were subsequently received ascorbate (6 g/kg) treatment two weeks later prior to LIE challenge. Mice were euthanized at 120 h after LIE, and serum and kidney tissues were analyzed.

### Plasmids, antibodies and reagents

The siRNA duplexes targeting PINK1, PARK2 and NIX were as previously described 17. The pGL3-ARE-luc vector was generated by inserting three copies of the antioxidant response element (ARE; 5'-GTGACAAAGCAATCCCGT GACAAAGCAATCCCGTGACAAAGCAATA-3') into pGL3-basic luciferase reporter plasmid. pLV-H1-SGIPZ-shNRF2 was constructed by inserting *NRF2* shRNA into lentiviral pLV-H1-SGIPZ vectors (119217, Addgene). Lentiviral shRNA targeting *Atg7* (TRCN0000092164) was obtained from the TRC library (Merck). Hemagglutinin (HA)-tagged NRF2 was constructed by subcloning mouse *NRF2* cDNA into pcDNA3.1-HA vector from Addgene (128034). pCDH-GFP-SerpinG1 plasmid was produced by subcloning mouse *SerpinG1* cDNA into the pCDH-MSCV-MCS-EF1ɑ-GFP-T2A-Puro vector (CD713B-1, System Biosciences, San Francisco, USA). AAV9-Ksp-GFP-shSerpinG1 plasmid was generated by subcloning SerpinG1 shRNA cassette into the adeno-associated virus 9 vector bearing the GFP-linked Ksp-cadherin promoter with oligonucleotides: AGCCAAGTGGAAGATAACATTTGTGAAGCCACAGATGAAA TGTTATCTTCCACTTGGCG. The siRNA duplexes against NRF2 and SerpinG1 were synthesized from GenePharma (Shanghai, China).

Fluorescently labeled antibodies for mF4/80 (BM8), miNOS (CXNFT), mARG1 (A1exF5), mCD206 (MR6F3) and mCD86 (GL1) were ordered from eBioscience^TM^ (San Diego, CA). Antibodies of iNOS (D6B6S), ARG1 (D4E3M^TM^), F4/80 (D4C8V), NRF2 (D1Z9C), phospho-ULK1_Ser555 (D1H4) and HSP90 (E289) used for immunofluorescence, chromatin immunoprecipitation, immunochemistry staining or western-blotting were from Cell Signaling Technology (Danvers, MA). Antibodies for DAB2 (E-11) and ATG7 (B-9) were obtained from Santa Cruz Biotechnology (Santa Cruz, CA). Both SVCT1 (ab236878) and SVCT2 (ab229802) Abs were from Abcam (Cambridge, MA, USA). Anti-SerpinG1 (NBP1-32478), anti-TNC (4C8MS), anti-SerpinB2 (NBP1-33188) and anti-VEGFC (NB110-61022) Abs were from Novus Biologicals (Colorado, US, USA). Anti-SOD2 (SAB5700729), anti-PINK1 (P0076) and anti-PARK2 (SAB2500749) Abs were from Sigma-Aldrich (St. Louis, MO). The neutralizing antibody to SerpinG1 (ab229209) was purchased from Abcam. Antibody for TOMM20 (11802-1-AP) and IGF1 (28530-1-AP) were from Proteintech Group, Inc. (Chicago, IL). Anti-NIX (bs-5798R) Ab was purchased from Biosynthesis (Beijing, China).

The* Escherichia coli* 0111: B4 LPS (L2630) and L-ascorbate (A4403) were ordered from Sigma-Aldrich (St. Louis, MO). Clodronate liposomes was from www.ClodronateLiposomes.org. Liensinine (HY-N0484) and CCCP (HY-100941) were purchased from Medchem Express (Monmouth Junction, New Jersey, USA). ELISA kits for mouse IL-1β (DY401), IL-18 (7625), IL-12 p70 (DY419), IL-10 (M1000B), CXCL-2 (MM200), GDF-15 (MGD150), TNF (DY410), CCL3 (MMA00), IL-4 (M4000B) and IFN-γ (MIF00) were from R&D Systems (Minneapolis, MN). QuantiChrom^TM^ assay kits for detecting serum creatinine (DICT-500) and blood urea nitrogen (DIUR-100) were from BioAssay Systems (Hayward, CA). ELISA kits for human and mouse SerpinG1 (SEA235Hu and SEA235Mu) were ordered from Wuhan USCN Business Co. (Wuhan, China).

### Cell culture

Human kidney proximal tubular epithelial HK-2 cells from American Type Culture Collection (Manassas, VA, USA) were cultured in RPMI-1640 (GIBCO, Carlsbad, CA) supplemented with 10% heat-inactivated FBS, 2 mM glutamine, 100 U/mL penicillin and 100 g/mL streptomycin in a humidified incubator with 5% CO_2_ at 37 ℃ as previously described^17^, ^20^, ^21^. SVCT-1 and/or -2 knockout (KO) murine renal tubular epithelial cells (RTECs) in which endogenous SVCT-1 and/or -2 were knocked out by CRISPR-Cas9 genome editing were generated and grown as previously described 17. In brief, renal cortices were dissected, sliced and transferred through 125 and 106 µm strainers, respectively. The tubular fragments were seeded in collagen-coated plates with Dulbecco's modified Eagle's medium/F12 (DMEM/F12), 10% heat inactivated FBS, and 1% L-glutamine at 37 °C in a humidified incubator equipped with 5% CO_2_. Commercial sgRNAs (sc-422995 and sc-424830) from Santa Cruz Biotechnology (Santa Cruz, CA) were utilized to knock out SVCT-1 and/or -2, and clones derived from the KO RTECs were obtained by serial dilutions in a 96-well plate.

To collect macrophages for reconstitution experiments, spleens harvested from PBS- or high-dose ascorbate (6 g/kg)-treated mice were passed through a 40 μm strainer (Fisher Scientific, Fair Lawn, NJ) to generate single-cell suspension in PBS. The non-adherent cells were removed three days later and the supernatant was discarded at day 6. The adherent splenic macrophages were then washed three times with Dulbecco's Ca^2+^/Mg^2+^-free PBS (DPBS, HyClone, Logan) and incubated with Cellstripper nonenzymatic cell dissociation solution (Mediatech, Manassas, VA) for 5 min. After being added to ice-cold complete RPMI-1640, macrophages were centrifuged at 350 g for 5 min and resuspended in media for subsequent experiments.

Bone marrow-derived macrophages (BMDMs) were isolated and as prepared under sterile conditions using femur of mice with the treatments as indicated. Briefly, bone marrow was flushed out using sterile phosphate-buffered saline (PBS) and passed through a 70 μm filter. Cells were washed with PBS, resuspended and maintained in six-well plates with DMEM medium containing 10% FBS and 100 U/mL penicillin and 100 mg/mL streptomycin at 37 ℃ in 5% CO_2_ at a humidified atmosphere. Non-attaching cells were removed six hours later and cells were allowed to differentiate over seven days with addition of 20 μg/mL macrophage colony-stimulating factor (M-CSF) every three days.

### Coculture experiments and conditioned medium collection

For coculture assays, BMDMs (0.5×10^5^) were plated in the lower chamber of a 12-well transwell apparatus with 0.4 μm pore size (Costar, Cambridge, MA) and the freshly prepared RTECs (1×10^6^) were then plated in upper chamber with single LPS (200 ng/mL) stimuli or LPS plus ascorbate (125 µmol/L) costimuli. For mitophagy blockade assays, RTECs were transfected with scrambled shRNA (Scr) and *Atg7* shRNA before being cocultured with BMDMs, respectively. For experimental settings against SerpinG1 secretion, RTECs were plated after transfection with siRNA duplexes targeting *SerpinG1*. The untransfected or control siRNA-transfected RTECs served as controls.

To collect the conditioned medium (CM), RTECs were plated in medium containing 10% FBS at identical densities and next day the mediums were replaced with equal volumes of low-serum medium containing 0.1% FBS. After culture for additional 24 h, the mediums were collected and centrifuged at 350 g for 10 min. The supernatants were then harvested in subsequent experiments. For mitophagy blockade assays, liensinine (50 µmol/L) was added into low-serum medium and replenished after 24 h. For the experiments with the SVCT-1 and/or -2 KO or ATG7-depleted RTECs, CM was collected as similarly as above-mentioned. For experiments involving SerpinG1 neutralization, the medium derived from RTECs expressing GFP-SerpinG1 was preincubated with anti-SerpinG1 Ab or appropriate control anti-IgG Ab with a concentration of 20 μg/mL for 2 h before being added into BMDMs cultures.

### Lentiviral transduction

Preparation of ATG7 shRNA particles was based on pLKO.1-puro vectors as described previously^17^. The NRF2 shRNA particles were produced by HEK293T cells that were transfected with lentiviral pLV-H1-SGIPZ vectors encoding *NRF2*-specific shRNA along with the packaging vectors psPAX2 (3 μg) and pMD2.G (1 μg). Medium was harvested at 48 h after package and RTECs (2×10^6^) were transduced by combining 0.5 mL of medium containing viral particle plus 2 mL of complete medium with polybrene at a final concentration of 8 μg/mL. Media were exchanged after 6 h and selection was performed using 2 μg/mL puromycin (Sigma Aldrich, St Louis, MO, USA) 18 h later.

### Flow cytometry analysis

The percentage of macrophages with M1 or M2 subtype were sorted by flow cytometry using antibodies against iNOS, ARG1, CD206 and CD86, respectively. In brief, BMDMs (5×10^5^) with or without LPS stimuli were underwent the indicated treatments, harvested and incubated with specific antibodies (1 μg) on ice for 30 min in darkness. The solutions were then washed twice by 2 mL cell staining buffer, centrifuged at 350 g for 5 min, fixed with 2% paraformaldehyde in the dark for 20 min, permeablized in 0.5% saponin and subjected to flow cytometry analyses.

Fluorescence activating cell sorter (FACS) data were analyzed in FlowJo v.10 (Ashland, OR) and the identities of macrophages were corroborated by anti-F4/80 antibody.

### Immunofluorescence and immunohistochemistry

Immunofluorescence (IF) and immunohistochemistry (IHC) stainings were performed on paraffin-embedded sections as previously reported 17, 20. For IF staining, 4 μm renal slides were baked for 120 min at 60 ℃, deparaffinized and rehydrated with serial passage through changes of xylene and graded alcohol. Antigen was retrieved at ethylene diamine tetraacetic acid (EDTA) antigen retrieval buffer (pH 8.0) and endogenous peroxidase was blocked by incubation of slides with 2% hydrogen peroxide (H_2_O_2_). After spontaneous fluorescence quenching, slides were blocked in 3% bovine serum albumin (BSA) in PBS and 0.25% Triton X-100 for 1 h at room temperature. Slides were incubated with the indicated primary antibodies in the blocking solution overnight at 4 ℃ and the following day with the Alexa Fluor^®^ 488 (Abcam; 1:500)- or Alexa Fluor^®^ 647 (Abcam; 1:500)-conjugated secondary antibodies for 30 min at room temperature. After extensive washing in PBS containing 0.25% Triton X-100, slides were coversliped with anti-fade mounting medium. Nuclei were counterstained with 4', 6-diamidino-2-phenylindole (DAPI) and images were captured on a Carl Zeiss 8 (Oberkochen, Germany) Axioimager Z1 microscope.

For IHC staining, slides were probed with the primary antibodies as indicated and diluted in BSA overnight at 4 °C, followed by washing three times with Tris-Buffered Saline and Tween 20 (TBST) and incubating with horseradish peroxidase-conjugated secondary antibodies diluted in BSA for 1 h at room temperature. Slides were washed again and signals were attained by Dako ChemMateTM Envision^TM^ Detetcion Kit (DaKo, Glostrup, Denmark) for 5 min at room temperature. The slides were counterstained with hematoxylin for 1 min and rinsed with warm tap water for 5 min. Images were obtained using an AxioVision Rel.4.6 computerized image-analysis system (Carl Zeiss).

### Mitochondrial function

Mitochondrial membrane potential (ΔΨm), cytosolic mtDNA, mitochondrial reactive oxygen species (mtROS) were measured by TMRM Mitochondrial Membrane Potential Assay Kit (BioVision, Milpitas, CA), real-time quantitative PCR (RT-qPCR) with the specific primers for cytochrome c oxidase 1 (COX1) and MitoSOX^TM^ Red (Thermo Fisher Scientific, Waltham, MA, USA) as previously described^17^. For TOMM20 staining, RTECs were cultured overnight on glass coverslips, fixed with 4% paraformaldehyde in PBS for 15 min and permeabilized with 0.2% Triton X-100. Cells were then blocked with 5% BSA and incubated with primary anti-TOMM20 Abs overnight at 4 °C. Next day, after being washed by PBS and Tween 20 (PBST) for three times, cells were incubated with secondary Abs for 1 h at room temperature and rinsed in PBST, followed by mounting with DAPI and analyses.

### Western blotting

Western-blotting analyses were carried out as previously described with some modifications^17^, ^20^-^22^. In brief, 30 µg of protein was loaded on the gel, separated by 10% sodium dodecyl sulfate (SDS)-polyacrylamide gel (PAGE) electrophoresis and transferred to a Immobilon^TM^ PVDF Transfer Membranes (Millipore Corporation, Billerica, MA). The membranes were then blocked by incubating with 5% BSA in Tris-buffered saline and 0.1% Tween 20. After blocking, membranes were probed overnight with the appropriate dilution of the primary antibodies and revealed with horseradish peroxidase (HRP)-conjugated secondary antibodies for 1 h at room temperature. The blots were developed by western chemiluminescent HRP Substrate Kit (PPLYGEN, Beijing, China) according to the manufacturer's protocol after washing.

### Enzyme-linked immunosorbent assay (ELISA)

Renal tissues were isolated from mice immediately after sacrifice, homogenized in 1:9 (w/v) normal saline with glass homogenizer for 1 min and centrifuged at 12, 000 rpm for 20 min. Protein concentration was determined by BCA protein assay kit (Thermo Fisher Scientific) with bovine serum albumin as a standard. The concentrations of IL-1β and IL-18 in supernatants of renal homogenate were measured by commercial ELISA kits (R&D Systems). The concentrations of IL-12, IL-10, CXCL-2, GDF15, TNF, CCL3, IL-4 and IFN-γ in cell cultures were determined by ELISA kits (R&D Systems) according to manufacturer's instructions, respectively. The absorbance was measured using the Epoch Microplate Spectrophotometer (Bio-Tek, Winooski, VT).

### Real-time quantitative reverse transcriptase-polymerase chain reaction (RT-qPCR)

RNA was extracted with TRIzol reagent (Invitrogen; 15596026) from the indicated RTECs. RNA was then reverse-transcribed into cDNA using the PrimeScript RT Reagent Kit (Takana, Dalian, China). RNA samples were subjected to quantitative PCR analyses using SYBR Green PCR Master Mix (Applied Biosystems). The expression of individual genes was calculated by a standard-curve method and was normalized to the expression of *GAPDH*. Gene-specific primers used in this study were listed in Supplemental Table 2.

### RNA-sequencing and gene set enrichment analysis (GSEA)

RNA was isolated from the renal tissues of the LIE-challenged *Atg7*^flox/flox^ and *Atg7*^ΔTE^ mice receiving high-dose ascorbate therapy by TRIzol reagent according to the manufacturer's instructions, respectively. Strand-specific cDNA libraries were constructed following a previously described protocol and were sequenced using an Illumina HiSeq6000 sequencer (OEbiotech, Shanghai, China) to generate dataset. The raw reads were processed by removing the adaptor reads and low-quality tags. Genes that were differentially expressed between renal tissues of the LIE-challenged *Atg7*^flox/flox^ and *Atg7*^ΔTE^ mice receiving high-dose ascorbate therapy were subjected to gene set enrichment analysis (GSEA), which was performed using the BROAD javaGSEA standalone version (http://www.broadinstitute.org/gsea/downloads.jsp) and the NRF2 target gene set collection (BROAD molecular signature database, MSigDbv4.0, http://www.broadinstitute.org/gsea/msigdb/index.jsp). The gene-set permutation mode and default setting of 1,000 permutations were used, and the metric for ranking genes in GSEA was set as Pearson's correlation analysis.

### Luciferase reporter assay

Luciferase reporter assay was performed as previously described 20, 21, 23, 24. The ARE-luciferase reporter plasmids were transfected into the indicated RTECs using Lipofectamine 3000 reagent (Invitrogen). Each of transfection was included the same amount of pRL-TK Renilla plasmid, which was employed to standardize transfection efficiency. At 48 h after transfection, the luciferase activities in cell lysates were determined with the luciferase assay system (Promega) using a BioTek Synergy2 Microplate reader (Bio-Tek) at wavelengths of 560 and 465 nm according to the manufacturer's guidelines.

### Chromatin immunoprecipitation (ChIP) assay

ChIP was performed with lysates prepared from the untransfected, empty vector- or HA-NRF2-transfected RTECs using the Epitect Chip OneDay kit (QIAGEN, 334471) as described previously 20, 22-24. In brief, the indicated RTECs were fixed with 1% formaldehyde for 15 min and quenched with 125 mM glycine. Chromatin was isolated by adding lysis buffer (50 mM Tris, 1% NP40, 150 mM NaCL, 0.01% SDS, 1.2 mM EDTA, 1 mM PMSF) and the DNA was sheared to an average length of 300-1000 bp. An aliquot of chromatin was pre-cleared with protein G agarose beads and genomic DNA regions were isolated with 2 μg of anti-IgG and anti-NRF2 antibodies, respectively. Protein-DNA complexes were eluted from the beads and incubated with RNase (QIAGEN) and proteinase K (Roche). Crosslinks were reversed by 300 mM NaCl and ChIP DNA was purified by phenol/chloroform extraction and ethanol precipitation. RT-qPCR was performed on the purified DNA fragments using mouse SerpinG1 primers:

Forward, 5'-CTCCTCCCTCAAAGCTGTGA-3';

Reverse, 5'-TCGGCCCATCTGTTCAATCT-3'.

### Statistical analysis

Survival differences between groups were calculated by log-rank test. Differences between two groups were evaluated by two-sided Student's *t* test. Other statistical analyses were performed by two-way ANOVA with Bonferroni correction using SPSS 20.0 for Windows (SPSS Inc, Chicago,IL, USA). *P* values less than 0.05 were considered significant and error bars indicated the standard deviation in all figures.

## Results

### Phenotypic conversion of macrophages instructs the anti-septic AKI efficacy of high-dose ascorbate

**Figure 1:**
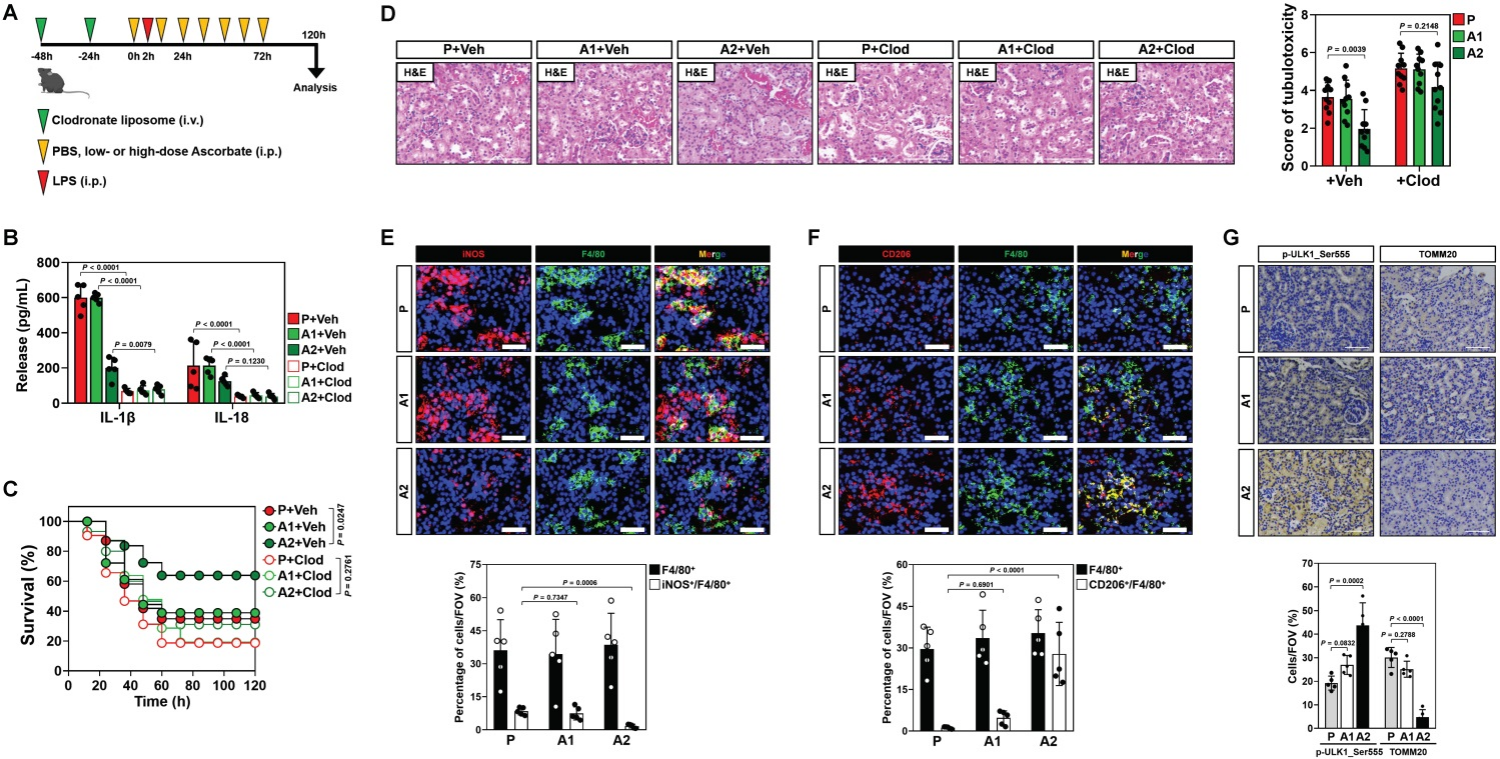
** Conversion of pro-inflammatory macrophages to anti-inflammatory phenotypes contributes to the anti-septic AKI efficacy of high-dose ascorbate therapy. (A)** Experimental scheme illustrating therapeutic regimen of high-dose ascorbate for LPS-induced endotoxemia (LIE) mice with clodronate liposomes pretreatment at the indicated time points. **(B)** Enzyme-linked immunosorbent assay (ELISA) measuring interleukin-1β (IL-1β) and interleukin-18 (IL-18) production in kidney homogenate of clodronate liposomes-pretreated mice with PBS (P), low- or high-dose ascorbate (A1 or A2) therapy upon LIE challenge (*n* = 5 per group). Data are expressed as mean ± s.d. Two-sided ANOVA with Bonferroni post hoc *t* test correction was used to calculate the *P* value. Veh, vehicle. Clod, clodronate liposomes. **(C)** Kaplan-Meier curves analyzing survivals of clodronate liposomes-pretreated mice with PBS (P), low- or high-dose ascorbate (A1 or A2) therapy upon LIE challenge (*n* > 12 mice per group). Log-rank t test was used to calculate the *P* value. **(D)** Representative images (left panel) and quantification (right panel) of H&E staining in renal sections from clodronate liposomes-pretreated mice with PBS (P), low- or high-dose ascorbate (A1 or A2) therapy upon LIE challenge (*n* = 10 per group). Data are expressed as mean ± s.d. Two-sided ANOVA with Bonferroni post hoc *t* test correction was used to calculate the *P* value. **(E-G)** Representative images (top panel) and quantification (bottom panel) of iNOS^+^/F4/80^+^, CD206^+^/F4/80^+^, p-ULK1_Ser555 and TOMM20 staining in renal sections from LIE mice with PBS (P), low- or high-dose ascorbate (A1 or A2) therapy (*n* = 5 per group). Data are expressed as mean ± s.d. Two-sided ANOVA with Bonferroni post hoc *t* test correction was used to calculate the *P* value. Scale bar: 50 μm and 100 μm.

In recent study, we showed that high-dose ascorbate confers tubular mitophagy to prevent septic AKI in murine models of LPS-induced endotoxemia (LIE) 17. We also observed that LIE mice receiving high-dose ascorbate therapy had reduced production of renal IL-1β and IL-18 (Figure S1A, B), two pro-inflammatory cytokines released mainly by macrophages 25. To explore the functional roles of macrophages in anti-septic AKI efficacy of high-dose ascorbate, we employed clodronate liposome to eliminate macrophages in mice (Figure 1A and Figure S1C). Administration of clodronate liposome effectively diminished production of renal IL-1β and IL-18, and impaired the ability of high-dose ascorbate to attenuate mortality and tubulotoxicity of LIE mice (Figure 1B-D), implicating that macrophages play key roles in the anti-septic AKI efficacy of high-dose ascorbate therapy.

To decipher whether and how high-dose ascorbate therapy leads to alterations of macrophages, we examined the phenotypes of infiltrated macrophages in renal sections of LIE mice, where F4/80 is a pan-macrophage marker while inducible nitric oxide synthase (iNOS) is alternatively expressed in the pro-inflammatory M1 macrophages and CD206 represents a productive marker of the anti-inflammatory M2 subtype. We observed a profound decrease in the inducible nitric oxide synthase (iNOS)^+^ macrophages but increase in the CD206^+^ macrophages from LIE mice receiving high-dose ascorbate therapy with comparable number of F4/80^+^ macrophages (Figure 1E, F), which were accompanied by an induction of tubular mitophagy as reflected by immunohistochemistry (IHC) staining for ULK1 phosphorylation at Ser555 and translocase of outer mitochondrial membrane 20 homolog (TOMM20) (Figure 1G). These results suggest that high-dose ascorbate simultaneously elicits tubular mitophagy and a switch from the pro-inflammatory M1 macrophages to anti-inflammatory M2 subtype during septic AKI.

To elucidate the role of macrophages phenotypic switch in the anti-septic AKI efficacy of high-dose ascorbate therapy, we isolated splenic macrophages from mice receiving PBS (P) or high-dose ascorbate (A2) injection and adoptively transferred them into the clodronate liposome-treated mice; the recipient mice were then subjected to LIE in the presence of high-dose ascorbate therapy (Figure S1D). Staining for F4/80 identified comparable proportion of macrophages among renal sections of LIE mice receiving macrophages transfer. However, renal sections of LIE mice receiving the P or A2 macrophages transfer and high-dose ascorbate therapy displayed less iNOS^+^ but more CD206^+^ population, in contrast to those receiving P or A2 macrophages transfer and PBS therapy. The proportion of neither iNOS^+^ nor CD206^+^ macrophages in LIE mice receiving A2 macrophages transfer differed significantly from those in LIE mice receiving P macrophages transfer (Figure S1E-G). The greatest tubulotoxicity was observed in the macrophages-eliminated mice receiving only either PBS or high-dose ascorbate therapy after LIE challenge. Although adoptive transfer of A2 macrophages partially protected the macrophages-eliminated LIE mice from tubulotoxicity to a similar extent as P macrophages did, mice receiving concomitant PBS therapy still developed septic AKI as compared with those receiving concomitant high-dose ascorbate therapy upon LIE challenge (Figure S1E, H). We detected a prominent induction of tubular mitophagy from renal sections of all LIE mice following high-dose ascorbate therapy regardless of adoptive transfer (Figure S1E, I). ELISA showed increased production of renal IL-1β and IL-18 in the macrophages-eliminated LIE mice receiving P or A2 macrophages transfer, which could be reversed by high-dose ascorbate therapy (Figure S1J, K). These results suggest that high-dose ascorbate-elicited phenotypic switch of macrophages governs the primary initiation of anti-septic AKI immunity, yet high-dose ascorbate by itself is insufficient to prime macrophages for the phenotypic switch during this process.

### High-dose ascorbate confers tubular mitophagy to boost anti-inflammatory macrophages during septic AKI

**Figure 2:**
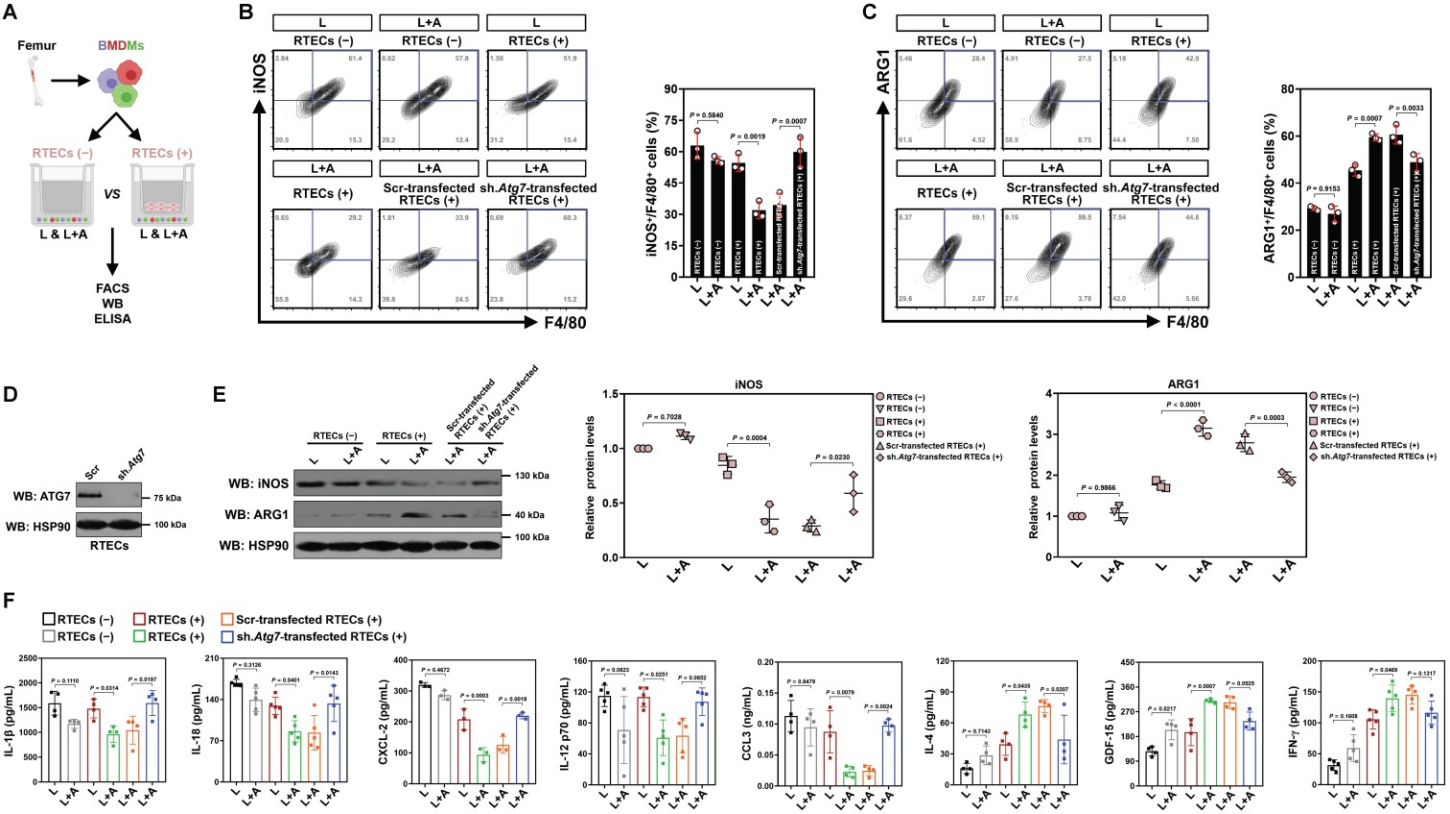
** Tubular mitophagy is instrumental for the high-dose ascorbate-boosted M2 macrophages *in vitro*. (A)** Experimental scheme depicting fluorescence-activated cell sorting (FACS), western-blotting and ELISA of the LPS-stimulated or LPS plus high-dose ascorbate-costimulated bone marrow-derived macrophages (BMDMs) with or without renal tubular epithelial cells (RTECs) coculture. **(B and C)** Representative FACS contour plots comparing iNOS^+^/F4/80^+^ and ARG1^+^/F4/80^+^ populations in the LPS-stimulated or LPS plus high-dose ascorbate (125 μmol/L)-costimulated BMDMs with the scrambled shRNA (Scr)- or *Atg7* shRNA (sh.*Atg7*)-transfected RTECs coculture (*n* = 3 per group). Data are expressed as mean ± s.d. Two-sided ANOVA with Bonferroni post hoc *t* test correction was used to calculate the *P* value. **(D)** Western-blotting analyses detecting ATG7 expression in RTECs transfected with scrambled shRNA (Scr) or *Atg7* shRNA (sh.*Atg7*). **(E)** Western-blotting analyses comparing abundance of iNOS and ARG1 protein in the LPS-stimulated or LPS plus high-dose ascorbate-costimulated BMDMs with the scrambled shRNA (Scr)- or *Atg7* shRNA (sh.*Atg7*)-transfected RTECs coculture (*n* = 3 per group). Data are expressed as mean ± s.d. Two-sided ANOVA with Bonferroni post hoc *t* test correction was used to calculate the *P* value. **(F)** ELISA evaluating secretion of interleukin-1β (IL-1β), interleukin-18 (IL-18), interleukin-12 (IL-12), C-X-C motif chemokine ligand-2 (CXCL-2), C-C motif chemokine ligand 3 (CCL3), growth polarization factor-15 (GDF-15), interleukin-4 (IL-4) and interferon-γ (IFN-γ) in the LPS-stimulated or LPS plus high-dose ascorbate-costimulated BMDMs with the scrambled shRNA (Scr)- or *Atg7* shRNA (sh.*Atg7*)-transfected RTECs coculture (*n* ≥ 3 per group). Data are expressed as mean ± s.d. Two-sided ANOVA with Bonferroni post hoc *t* test correction was used to calculate the *P* value.

We next enrolled *in vitro* cellular models to see if high-dose ascorbate directly boosts M2 macrophages or does so in a context-dependent manner. In this setting, bone marrow-derived macrophages (BMDMs) were stimulated with LPS or costimulated with LPS plus high-dose ascorbate in the presence or absence of freshly prepared renal tubular epithelial cells (RTECs) coculture (Figure 2A). We observed no changes in either iNOS^+^ or ARG1^+^ population among different groups before and after high-dose ascorbate exposure alone, while the high-dose ascorbate-exposed BMDMs tended to differentiate toward M2-subtype when they were cocultured with RTECs upon LPS stimuli (Figure 2B, C). Nonetheless, depleting *Atg7* of RTECs by lentiviral short hairpin RNA (shRNA) before coculture blunted the ability of high-dose ascorbate to boost BMDMs to differentiate into M2-subtype under the same conditions (Figure 2B-E). Upon LPS stimuli, BMDMs with the *Atg7* shRNA (sh.*Atg7*)-transfected RTECs coculture secreted more IL-1β, IL-18, CXCL-2 and CCL3 but less IL-4 than those with the scrambled shRNA (Scr)-transfected RTECs coculture following high-dose ascorbate exposure (Figure 2F). Conditioned medium (CM) derived from the *Atg7* shRNA-transfected RTECs with LPS plus high-dose ascorbate costimuli failed to boost M2 macrophages (Figure S2A-C). Similar results were yielded in the LPS-stimulated, high-dose ascorbate-exposed BMDMs cocultured with RTECs that were pretreated with the mitophagy inhibitor liensinine. BMDMs barely differentiated to M2-subtype when incubating them with CM from SVCT-1 and -2 knockout (KO) RTECs costimulated with LPS plus high-dose ascorbate (Figure S2D, E). Neither single SVCT-1 or -2 KO nor combined KO of SVCT-1 and -2 affected M1/M2 phenotypes of BMDMs stimulated with LPS (Figure S2F). These data strongly suggest that tubular mitophagy is crucial for the high-dose ascorbate-boosted M2 macrophages in response to inflammatory stress.

**Figure 3:**
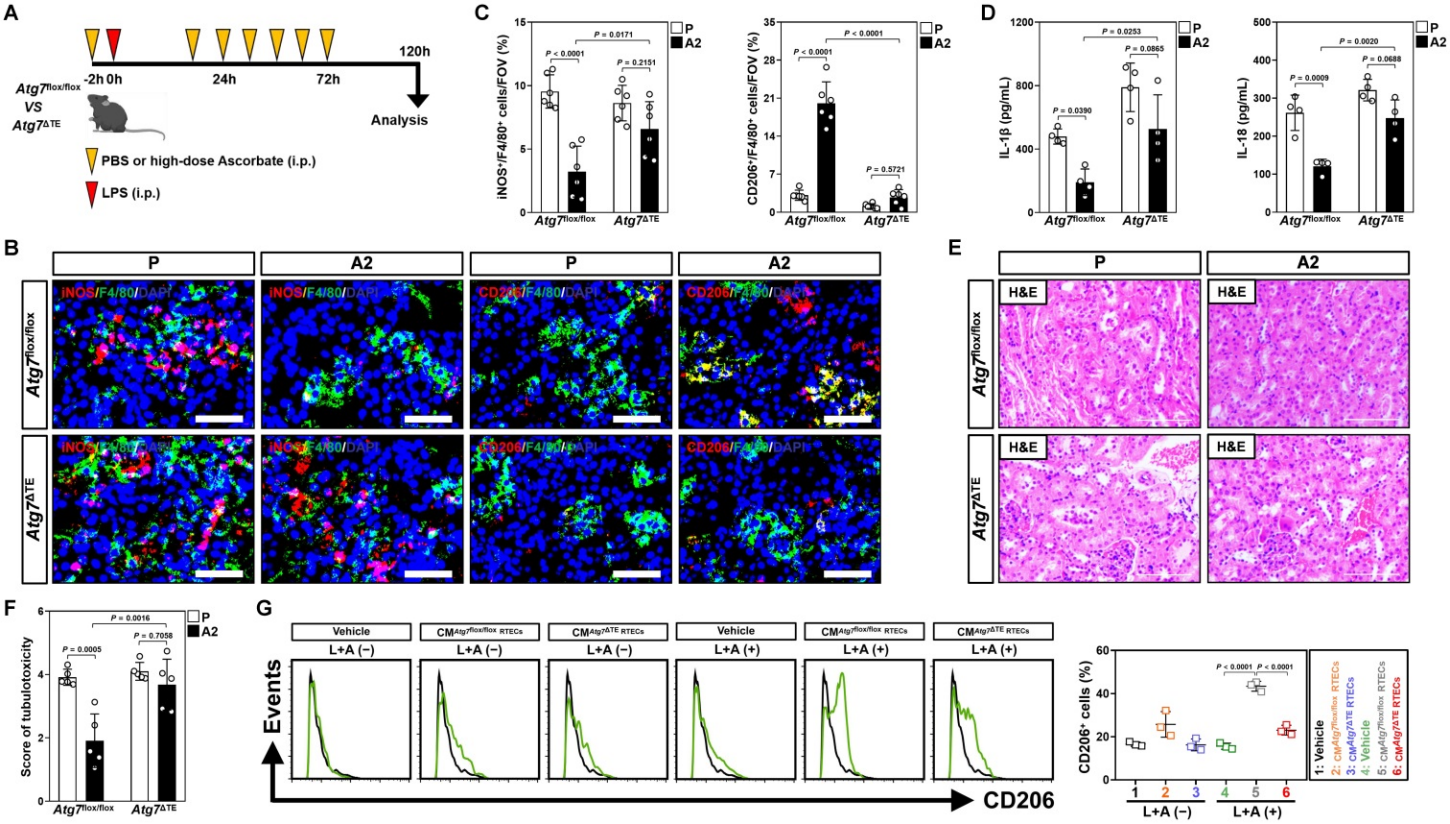
** Tubular epithelium-specific ATG7 ablation abrogates polarization of M2 macrophages and the anti-septic AKI effects by high-dose ascorbate therapy *in vivo*. (A)** Experimental scheme revealing therapeutic regimen of high-dose ascorbate for *Atg7*^flox/flox^ versus *Atg7*^ΔTE^ mice subjected to LPS-induced endotoxemia (LIE) at the indicated time points. **(B and C)** Representative images and quantification of iNOS^+^/F4/80^+^ and CD206^+^/F4/80^+^ staining in renal sections from *Atg7*^flox/flox^ and *Atg7*^ΔTE^ mice receiving PBS (P) or high-dose ascorbate (A2) therapy upon LIE challenge (*n* = 6 per group). Data are expressed as mean ± s.d. Two-sided ANOVA with Bonferroni post hoc *t* test correction was used to calculate the *P* value. Scale bar: 50 μm. **(D)** Enzyme-linked immunosorbent assay (ELISA) measuring interleukin-1β (IL-1β) and interleukin-18 (IL-18) production in kidney homogenate of *Atg7*^flox/flox^ and *Atg7*^ΔTE^ mice receiving PBS (P) or high-dose ascorbate (A2) therapy upon LIE challenge (*n* = 4 per group). Data are expressed as mean ± s.d. Two-sided ANOVA with Bonferroni post hoc *t* test correction was used to calculate the *P* value. **(E and F)** Representative images and quantification of H&E staining in renal sections from *Atg7*^flox/flox^ and *Atg7*^ΔTE^ mice receiving PBS (P) or high-dose ascorbate (A2) therapy upon LIE challenge (*n* = 5 per group). Data are expressed as mean ± s.d. Two-sided ANOVA with Bonferroni post hoc *t* test correction was used to calculate the *P* value. Scale bar: 100 μm. **(G)** Representative FACS histograms testing CD206^+^ populations in BMDMs incubated with conditioned medium (CM) from RTECs of *Atg7*^flox/flox^ and *Atg7*^ΔTE^ mice under LPS plus high-dose ascorbate-costimulated circumstances, respectively (*n* = 3 per group). Data are expressed as mean ± s.d. Two-sided ANOVA with Bonferroni post hoc *t* test correction was used to calculate the *P* value.

To validate whether the contribution of tubular mitophagy in M2 macrophages polarization conferred by high-dose ascorbate could be recapitulated *in vivo*, mice lacking ATG7 expression in tubular epithelium of kidney (*Atg7*^ΔTE^) were subjected to LIE in the presence or absence of high-dose ascorbate therapy (Figure 3A and Figure S3A). Corresponding to the defects in tubular mitophagy as reflected by immunohistochemical staining for p62 and transmission electron microscopy (TEM) for mitophagosomes, examination of renal sections from *Atg7*^ΔTE^ mice receiving high-dose ascorbate therapy identified increased iNOS^+^/F4/80^+^ but decreased CD206^+^/F4/80^+^ macrophages as compared to the control *Atg7*^flox/flox^ littermates upon LIE challenge (Figure 3B, C and Figure S3B). Further ELISA showed significant upregulation of renal IL-1β and IL-18 in *Atg7*^ΔTE^ mice with LIE in comparison to those in *Atg7*^flox/flox^ mice with LIE after high-dose ascorbate therapy (Figure 3D), proposing that tubular mitophagy is involved in the high-dose ascorbate-boosted M2 macrophages for the anti-inflammatory immune responses during septic AKI*.* The *Atg7*^flox/flox^ mice receiving high-dose ascorbate therapy showed resistance to the LIE-caused tubulotoxicity, whereas *Atg7*^ΔTE^ mice that received high-dose ascorbate therapy did not (Figure 3E, F). In line with the findings described for tubulotoxicity, *Atg7*^flox/flox^ littermates with high-dose ascorbate therapy had statistically significant lower levels of serum creatinine (Scr) and blood urea nitrogen (BUN) than those without, whereas such phenomena were not observed in *Atg7*^ΔTE^ mice (Figure S3C, D). The elevation in Scr and BUN—presumably due to exacerbated tubulotoxicity―was accompanied by a significant survival inferiority in *Atg7*^ΔTE^ mice receiving high-dose ascorbate therapy (Figure S3E). Macrophages elimination had negligible effects on tubulotoxicity of *Atg7*^ΔTE^ mice receiving high-dose ascorbate therapy (Figure S3F), indicating that the tubular mitophagy-boosted anti-inflammatory macrophages were responsible for the anti-septic AKI efficacy of high-dose ascorbate. The instrumental role of tubular mitophagy in the high-dose ascorbate-boosted M2 macrophages under inflammatory stress was further corroborated in BMDMs incubating with CM from the LPS plus high-dose ascorbate-costimulated *Atg7*^ΔTE^ RTECs, which were polarized into M2-subtype to a much lesser degree than BMDMs incubated with CM from the costimulated *Atg7*^flox/flox^ RTECs (Figure 3G and Figure S3G, H). Thus, high-dose ascorbate therapy elicits tubular mitophagy, thereby boosting the anti-inflammatory M2 macrophages to prevent septic AKI.

### High-dose ascorbate favors tubular secretion of SerpinG1 in a mitophagy-dependent fashion under inflammatory stress

**Figure 4:**
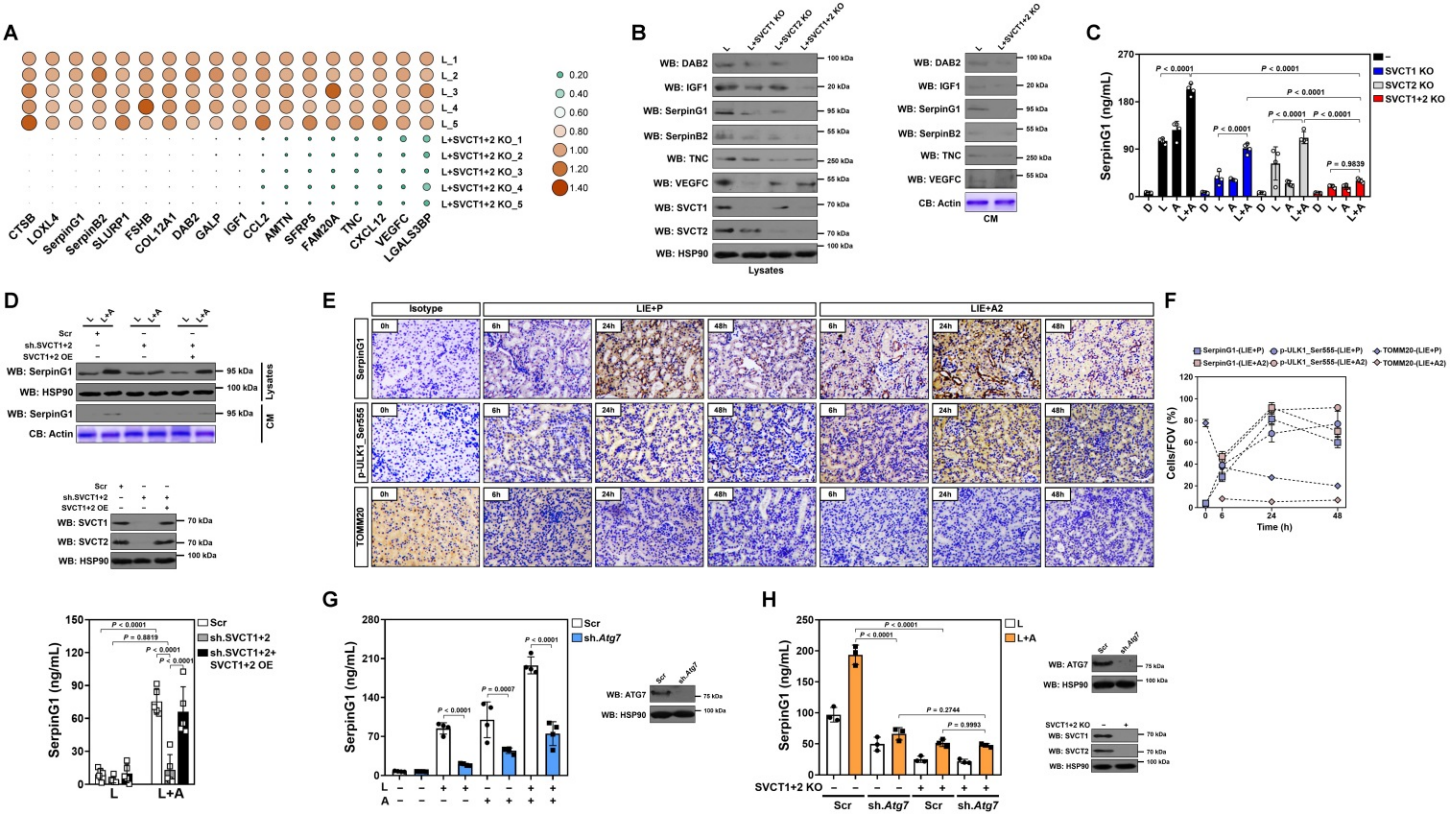
** High-dose ascorbate favors tubular secretion of SerpinG1 in a mitophagy-dependent fashion under inflammatory stress. (A)** Heatmap showing levels of downregulated genes encoding secreted proteins in SVCT-1 and -2 knockout (KO) RTECs with LPS stimuli (*n* = 5 per group). L, LPS. **(B)** Western-blotting analyses comparing abundance of DAB2, IGF1, SerpinG1, SerpinB2, TNC and VEGFC protein in cell lysates or conditioned medium (CM) from SVCT-1 and/or -2 knockout (KO) RTECs with LPS stimuli. CB: coomassie blue. **(C)** ELISA testing secretion of SerpinG1 in SVCT-1 and/or -2 knockout (KO) RTECs with LPS and/or high-dose ascorbate costimuli (*n* = 4 per group). Data are expressed as mean ± s.d. Two-sided ANOVA with Bonferroni post hoc *t* test correction was used to calculate the *P* value. **(D)** Western-blotting analyses and ELISA determining abundance and secretion of SerpinG1 in cell lysates or conditioned medium (CM) from SVCT-1 and -2 shRNA-transfected HK-2 cells with LPS and/or high-dose ascorbate costimuli in the presence or absence of reconstituted SVCT-1 plus -2 expression (*n* = 5 per group). Data are expressed as mean ± s.d. Two-sided ANOVA with Bonferroni post hoc *t* test correction was used to calculate the *P* value. OE: overexpression. **(E and F)** Representative images and quantification of immunohistochemistry (IHC) staining for SerpinG1 and p-ULK1_Ser555 in renal sections from LIE mice receiving PBS (P) or high-dose ascorbate (A2) therapy at the indicated times (*n* = 3 per group). Data are expressed as mean ± s.d. Scale bar: 100 μm. **(G)** ELISA evaluating secretion of SerpinG1 in the LPS and/or high-dose ascorbate-costimulated RTECs with scrambled shRNA (Scr) or *Atg7* shRNA (sh.*Atg7*) transfection (*n* = 4 per group). Data are expressed as mean ± s.d. Two-sided ANOVA with Bonferroni post hoc *t* test correction was used to calculate the *P* value. **(H)** ELISA assessing secretion of SerpinG1 in the LPS and high-dose ascorbate-costimulated RTECs in the presence or absence of SVCT-1 plus -2 KO and/or *Atg7* shRNA (sh.*Atg7*) transfection (*n* = 3 per group). Data are expressed as mean ± s.d. Two-sided ANOVA with Bonferroni post hoc *t* test correction was used to calculate the *P* value.

To surmise the factor directing M2 macrophage polarization in tubular cells, we examined putative modulatory factors that exhibit a ≤ -2.0-fold change in SVCT-1 and -2 KO RTECs under LPS stimuli using a secreted protein database^26^ (Table S1). Double KO of SVCT-1 and -2 downregulated mRNA and protein expression of DAB2, IGF1, SerpinG1, SerpinB2, TNC and VEGFC, with SerpinG1 showing a dramatic reduction in CM (Figure 4A, B and Figure S4A). High-dose ascorbate substantially increased SerpinG1 secretion in the LPS-stimulated RTECs but could no longer to do so when SVCT-1 and -2 had been knocked out (Figure 4C). Ascorbate withdrawal tended to attenuate SerpinG1 secretion despite such attenuation did not reach statistical significance (Figure S4B). We also evaluated the effects of ascorbate in SerpinG1 through the LPS plus high-dose ascorbate-costimulated human kidney proximal tubular epithelial HK-2 cells and observed reduced mRNA expression, protein levels and secretion of SerpinG1 after transfecting them with shRNA duplexes targeting SVCT-1 and -2. However, reconstituted expression of SVCT-1 and -2 restored SerpinG1 to the levels as seen in the costimulated cells with scrambled shRNA (Scr) transfection (Figure 4D and Figure S4C, D). These data suggest that tubular secretion of SerpinG1 is a common feature in response to high-dose ascorbate under inflammatory stress.

In order to delineate the relationship between tubular mitophagy and SerpinG1 secretion during septic AKI, we examined SerpinG1 variations by immunohistochemical staining and observed increased intensity of SerpinG1 in renal sections of LIE mice relative to their counterparts (Figure 4E). In accordance with these IHC data and our previous findings, the levels of ULK1 Ser555 phosphorylation also intensified, suggesting that mitophagy is associated with tubular SerpinG1 production in pathogenesis of septic AKI. Of note, high-dose ascorbate therapy further enhanced SerpinG1 intensity to an extent corresponding to the elevation of ULK1 Ser555 phosphorylation (Figure 4E, F). Increased protein levels of SerpinG1 were also observed in renal tissues from *Atg7*^flox/flox^ mice as compared with those from the *Atg7*^ΔTE^ murine models of LIE (Figure S4E). To validate whether tubular mitophagy maintains SerpinG1 secretion, we assessed *Atg7*^ΔTE^ RTECs stimulated by LPS and found that ATG7 deficiency led to a great reduction in mRNA expression and secretion of SerpinG1 (Figure S4F, G). Given PINK1/PARK2 and BNIP3/NIX cascades regulate various aspects of mitophagy 27, we investigated impact of the two mitophagy pathways on SerpinG1. For this purpose, RTECs were transfected with siRNA targeting PINK1, PARK2 and NIX, respectively, and then stimulated with LPS. Silencing PINK1 or PARK2, but not NIX, abolished SerpinG1 secretion of RTECs stimulated by LPS (Figure S4H). Treatment with OXPHOS uncoupler CCCP 28 resulted in increased protein secretion of SerpinG1 in the LPS-stimulated HK-2 cells (Figure S4I). These data collectively suggest that the PINK1/PARK2-mediated mitophagy is required for the high-dose ascorbate-inducible SerpinG1 secretion under inflammatory stress.

To understand whether mitophagy is a direct effect resulting from ascorbate uptake of tubular cells or secondary to tubular SerpinG1 secretion, we stimulated RTECs *in vitro*. Recombinant SerpinG1 (rSerpinG1) treatment did not boost mitophagy in the LPS-stimulated RTECs, while exposure to high-dose ascorbate did (Figure S4J). Upon LPS stimuli, the siRNA-mediated silencing of SerpinG1 had minimal effect on high-dose ascorbate-inducible mitophagy (Figure S4K), indicating that SerpinG1 serves a downstream effector responsible for tubular mitophagy mediated by ascorbate uptake under inflammatory stress.

Additional evidence that tubular mitophagy facilitates SerpinG1 secretion was obtained from the *Atg7* shRNA-transfected RTECs, which released much less SerpinG1 than the parental cells did following high-dose ascorbate exposure upon LPS stimuli (Figure 4G). High-dose ascorbate-induced SerpinG1 secretion in the LPS-stimulated RTECs became implicit when mitophagy was blocked by liensinine (Figure S4L). Double KO of SVCT-1 and -2 in the *Atg7*-depleted RTECs showed no additive effects in impeding the high-dose ascorbate-inducible SerpinG1 secretion upon LPS stimuli (Figure 4H), implicating that ascorbate uptake and mitophagy might act in the same pathway to induce tubular SerpinG1 secretion under inflammatory stress.

### NRF2 transactivation contributes to the high-ascorbate-inducible SerpinG1 secretion mediated by tubular mitophagy

**Figure 5:**
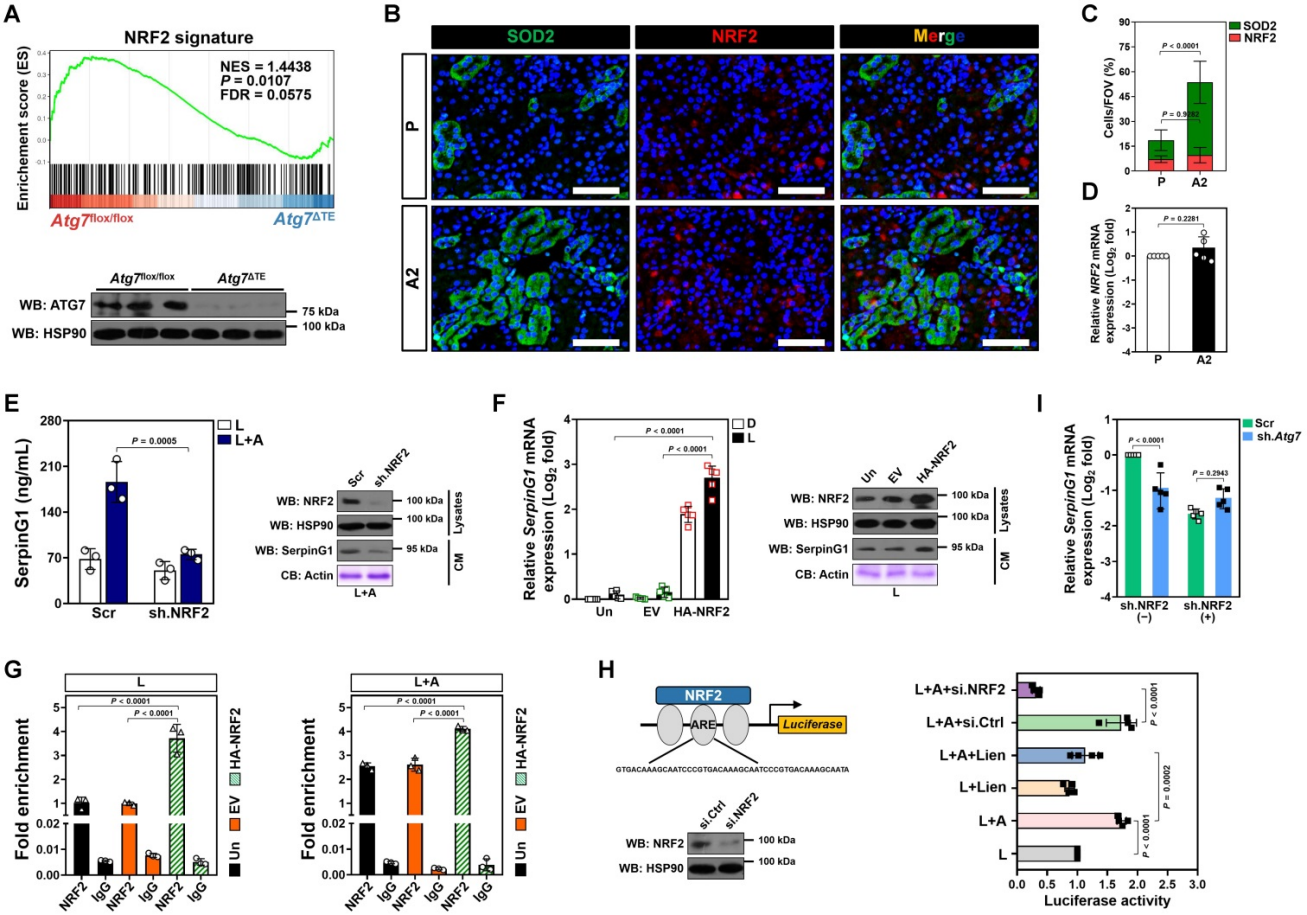
** NRF2 transactivation contributes to the high-ascorbate-inducible SerpinG1 secretion mediated by tubular mitophagy. (A)** Top panel: GSEA comparing correlation between renal transcriptomics of renal tissues from the LIE-challenged *Atg7*^flox/flox^ or *Atg7*^ΔTE^ mice receiving high-dose ascorbate therapy and NRF2 gene signature. Bottom panel: western-blotting analyses assessing abundance of SerpinG1 in renal tissues from *Atg7*^flox/flox^ and *Atg7*^ΔTE^ mice (*n* = 3 per group). **(B and C)** Representative images and quantification of SOD2^+^ and NRF2^+^ staining in renal sections from LIE mice with PBS or high-dose ascorbate therapy (*n* = 7 per group). Data are expressed as mean ± s.d. Two-sided Student's *t* test was used to calculate the *P* value. Scale bar: 50 μm. **(D)** RT-qPCR analysis assessing mRNA expression of *SerpinG1* in renal tissues from LIE mice with PBS or high-dose ascorbate therapy (*n* = 5 per group). Data are expressed as mean ± s.d. Experiments were performed five times, each with quantitative RT-PCR in technical duplicate and real-time values were normalized to glyceraldehyde 3-phosphate dehydrogenase (GAPDH). Two-sided Student's *t* test was used to calculate the *P* value. **(E)** Left panel: ELISA measuring secretion of SerpinG1 in the LPS-stimulated or LPS plus high-dose ascorbate-costimulated RTECs with NRF2.shRNA (sh.NRF2) transfection (*n* = 3 per group). Data are expressed as mean ± s.d. Two-sided ANOVA with Bonferroni post hoc *t* test correction was used to calculate the *P* value. Right panel: western-blotting analyses detecting levels of SerpinG1 in conditioned medium (CM) from LPS plus high-dose ascorbate-costimulated RTECs with NRF2.shRNA (sh.NRF2) transfection. **(F)** Left panel: RT-qPCR analysis evaluating mRNA expression of *SerpinG1* in the LPS-stimulated RTECs in the presence of HA-tagged NRF2 expression (*n* = 5 per group). Data are expressed as mean ± s.d. Two-sided ANOVA with Bonferroni post hoc *t* test correction was used to calculate the *P* value. Un: untransfected; EV: empty vector. Right panel: western-blotting analyses examining levels of SerpinG1 in conditioned medium (CM) from the LPS-stimulated RTECs in the presence of HA-tagged NRF2 expression. **(G)** ChIP analyses for NRF2 binding at *SerpinG1* promoter in the LPS-stimulated or LPS plus high-dose ascorbate-costimulated RTECs with HA-tagged NRF2 expression (*n* = 3 per group). Data are expressed as mean ± s.d. Two-sided ANOVA with Bonferroni post hoc *t* test correction was used to calculate the *P* value. **(H)** Luciferase assays of pGL3-ARE-luc activity in the LPS-stimulated or LPS plus high-dose ascorbate-costimulated HK-2 cells with or without liensinine (50 µmol/L) treatment (*n* = 4 per group). Data are expressed as mean ± s.d. Two-sided ANOVA with Bonferroni post hoc *t* test correction was used to calculate the *P* value. **(I)** RT-qPCR analysis evaluating mRNA expression of *SerpinG1* in the LPS plus high-dose ascorbate-costimulated RTECs transfected with ATG7 shRNA (sh.*Atg7*) in the presence or absence of NRF2 depletion (*n* = 5 per group). Data are expressed as mean ± s.d. Two-sided ANOVA with Bonferroni post hoc *t* test correction was used to calculate the *P* value.

Nuclear factor-erythroid 2 related factor 2 (NRF2)—activation of which can confer renal protection—serves as a potential therapeutic target in sepsis^29^, ^30^. Gene set enrichment analysis (GSEA) of renal tissues transcriptomics confirmed compromised NRF2 signalling in the LIE *Atg7*^ΔTE^ mice receiving high-dose ascorbate therapy (Figure 5A). Immunofluorescence staining of renal sections from LIE mice receiving high-dose ascorbate therapy revealed increased staining intensity of superoxide dismutase 2 (SOD2), an anti-oxidant enzyme transcriptionally activated by NRF2, as compared to those from LIE mice that were received PBS therapy (Figure 5B), while the staining intensity and mRNA expression of NRF2 were barely affected following high-dose ascorbate therapy (Figure 5B-D). These data suggest that high-dose ascorbate might induce mitophagy to transactivate NRF2 during septic AKI.

To unveil if there was a causal linkage between ascorbate uptake, NRF2 transactivation and SerpinG1 secretion under inflammatory stress, we depleted NRF2 in the LPS plus high-dose ascorbate-costimulated RTECs using shRNA and found that such setting readily ameliorated secretion of SerpinG1, reminiscent to that caused by ATG7 depletion (Figure 5E). A similar pattern was observed in the costimulated RTECs with alkaloid trigonelline 31 (Trig., an independent NRF2 chemical inhibitor) treatment (Figure S5A). Ectopic expression of hemagglutinin (HA)-tagged NRF2 into the LPS-stimulated RTECs eventually increased mRNA expression and secretion of SerpinG1 (Figure 5F and Figure S5B). Chromatin immunoprecipitation (ChIP) assay demonstrated that NRF2 preferentially bind to promoter of *SerpinG1* gene after high-dose ascorbate exposure (Figure 5G and Figure S5C), which reinforces the concept that NRF2 transcriptionally upregulates SerpinG1.

NRF2 is a transcription factor that exerts its biological function mainly by binding to the antioxidant response element (ARE) region of target gene promoters 32. To evaluate whether ascorbate influences transcriptional activity of NRF2 through mitophagy under inflammatory stress, we constructed a pGL3 luciferase reporter vector containing ARE and induced exogenous expression in HK-2 cells. Ascorbate exposure significantly enhanced the pGL3-ARE-luc activity upon LPS stimuli, which was blocked after mitophagy inhibition using liensinine (Figure 5H). ATG7 depletion failed to reduce mRNA expression and secretion of SerpinG1 when NRF2 had been depleted from cell lysates (Figure 5I and Figure S5D). These data imply that NRF2 functions as a downstream target of mitophagy to regulate the high-dose ascorbate-inducible SerpinG1 transcription in tubular cells under inflammatory stress.

### Tubular SerpinG1 perpetuates anti-inflammatory macrophages and thereby prevents septic AKI

**Figure 6:**
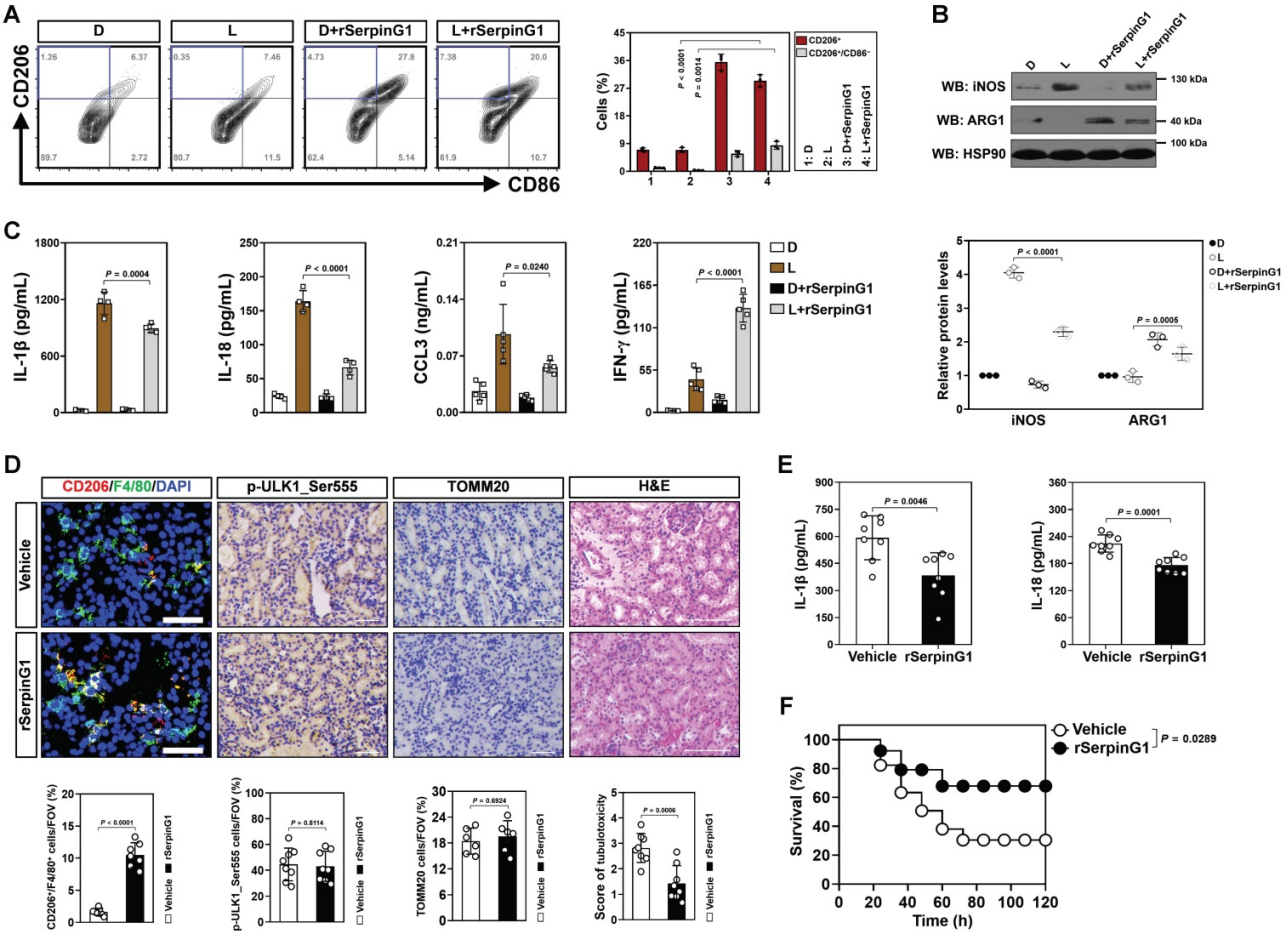
** SerpinG1 perpetuates anti-inflammatory macrophages and prevents septic AKI. (A)** Representative contour plots (left panel) and quantification (right panel) of FACS assessing CD206^+^ and CD206^+^/CD86^-^ populations in the LPS-stimulated or LPS plus rSerpinG1-costimulated BMDMs (*n* = 3 per group). Data are expressed as mean ± s.d. Two-sided ANOVA with Bonferroni post hoc *t* test correction was used to calculate the *P* value. D, DMSO. **(B)** Western-blotting analyses (top panel) and quantification (bottom panel) measuring abundance of iNOS and ARG1 protein in LPS-stimulated or LPS plus rSerpinG1-costimulated BMDMs (*n* = 3 per group). **(C)** ELISA assessing secretion of interleukin-1β (IL-1β), interleukin-18 (IL-18), C-C motif chemokine ligand 3 (CCL3) and interferon-γ (IFN-γ) in the LPS-stimulated or LPS plus rSerpinG1-costimulated BMDMs (*n* = 4 per group). Data are expressed as mean ± s.d. Two-sided ANOVA with Bonferroni post hoc *t* test correction was used to calculate the *P* value. **(D)** Representative images (top panel) and quantification (bottom panel) of CD206^+^/F4/80^+^, p-ULK1_Ser555 and H&E staining in renal sections from LPS-induced endotoxemia (LIE) mice receiving rSerpinG1 (800 μg) administration (*n* = 8 per group). Data are expressed as mean ± s.d. Two-sided Student's *t* test was used to calculate the *P* value. Scale bar: 50 μm and 100 μm. **(E)** ELISA comparing interleukin-1β (IL-1β) and interleukin-18 (IL-18) production in kidney homogenate of LPS-induced endotoxemia (LIE) mice receiving rSerpinG1 administration (*n* = 8 per group). Two-sided Student's *t* test was used to calculate the *P* value. **(F)** Kaplan-Meier curves determining survivals of LPS-induced endotoxemia (LIE) mice receiving rSerpinG1 administration (*n* ≥ 12 mice per group). Log-rank t test was used to calculate the *P* value.

The anti-inflammatory macrophages polarization and increased tubular SerpinG1 secretion by high-dose ascorbate-inducible mitophagy prompted us to pursue the functional role of SerpinG1 in macrophages. To approach this, BMDMs were treated with rSerpinG1 in the presence of LPS stimuli. rSerpinG1 increased CD206^+^/CD86^-^ population in the LPS-stimulated BMDMs, which manifested more CD206^-^/CD86^+^ population than the DMSO-stimulated BMDMs (Figure 6A). The protein levels of iNOS were decreased, while ARG1 levels were increased in the LPS-stimulated BMDMs after treating them with rSerpinG1 (Figure 6B). The LPS-stimulated BMDMs with rSerpinG1 treatment also secreted less IL-1β, IL-18 and CCL3 but more IFN-γ than those without (Figure 6C). These results demonstrate that SerpinG1 directs anti-inflammatory macrophages polarization in response to inflammatory stress.

To clarify the role of SerpinG1 in tubular cell-macrophage communications, we engineered RTECs to stably express GFP-tagged SerpinG1. CM derived from GFP-SerpinG1 transfectants enriched SerpinG1 protein and more efficiently perpetuated CD206^+^/CD86^-^ macrophages, reduced iNOS protein expression, elevated ARG1 levels as well as decreased IL-1β, IL-18 and CCL3 but increased IFN-γ secretion than CM derived from GFP transfectants upon LPS stimuli (Figure S6A-E). Neutralization of SerpinG1 with a specific antibody perturbed the ability of GFP-SerpinG1 CM to perpetuate M2 macrophage. Incubation with the high-dose ascorbate-exposed RTECs polarized the LPS-stimulated BMDMs to M2 subtype, but this effect was absent when incubating them with the exposed RTECs that were transfected with SerpinG1 siRNA (Figure S6F, G). Thus, the high dose ascorbate-inducible tubular SerpinG1 secretion is an important mediator for M2 macrophage polarization under inflammatory stress.

To further confirm the contributing role of SerpinG1 in M2 macrophage polarization during septic AKI, we administered rSerpinG1 to mice by tail-vein injection after LIE challenge. Renal sections of LIE mice receiving rSerpinG1 administration contained more CD206^+^/F4/80^+^ macrophages than those of LIE mice without administration, whereas the proportion of p-ULK1_Ser555-positive cells were comparable between the two groups (Figure 6D). The rSerpinG1-administered LIE mice exhibited significantly compromised tubulotoxicity, diminished production of renal IL-1β and IL-18 as well as prolonged survival duration in comparison with the control counterparts (Figure 6D-F). These results indicate that SerpinG1 perpetuates anti-inflammatory macrophages and prevents septic AKI.

To dissect the involvement of macrophages in the anti-septic AKI effects of SerpinG1, clodronate liposome was utilized *in vivo*. We observed that macrophages elimination dramatically impaired tubular protection and survival improvement by rSerpinG1 administration in LIE mice (Figure S6H, I). Therefore, macrophages may play an important role in the anti-septic AKI effects of SerpinG1. Together, our results reinforce that tubular SerpinG1 perpetuates the anti-inflammatory macrophage polarization and thereby prevents septic AKI.

### SerpinG1 is required for the anti-septic AKI efficacy of high-dose ascorbate

The induction of tubular mitophagy and SerpinG1 secretion by high-dose ascorbate therapy raised the question whether SerpinG1 participates in the suppressive role of mitophagy against septic AKI. To address this issue, rSerpinG1 was administered to the *Atg7*^ΔTE^ LIE mice receiving high-dose ascorbate therapy. Administering rSerpinG1 to the *Atg7*^ΔTE^ mice subjected to LIE resulted in increased magnitude of CD206^+^/F4/80^+^ macrophages in renal sections that were comparable to those in the *Atg7*^flox/flox^ mice subjected to LIE (Figure S7A). Administration of rSerpinG1 substantially reversed the deterioration in tubulotoxicity of the LIE-challenged *Atg7*^ΔTE^ mice (Figure S7A). The elevation in renal IL-1β production by tubular epithelium-specific *Atg7* ablation was also abrogated after rSerpinG1 administration (Figure S7B).

**Figure 7:**
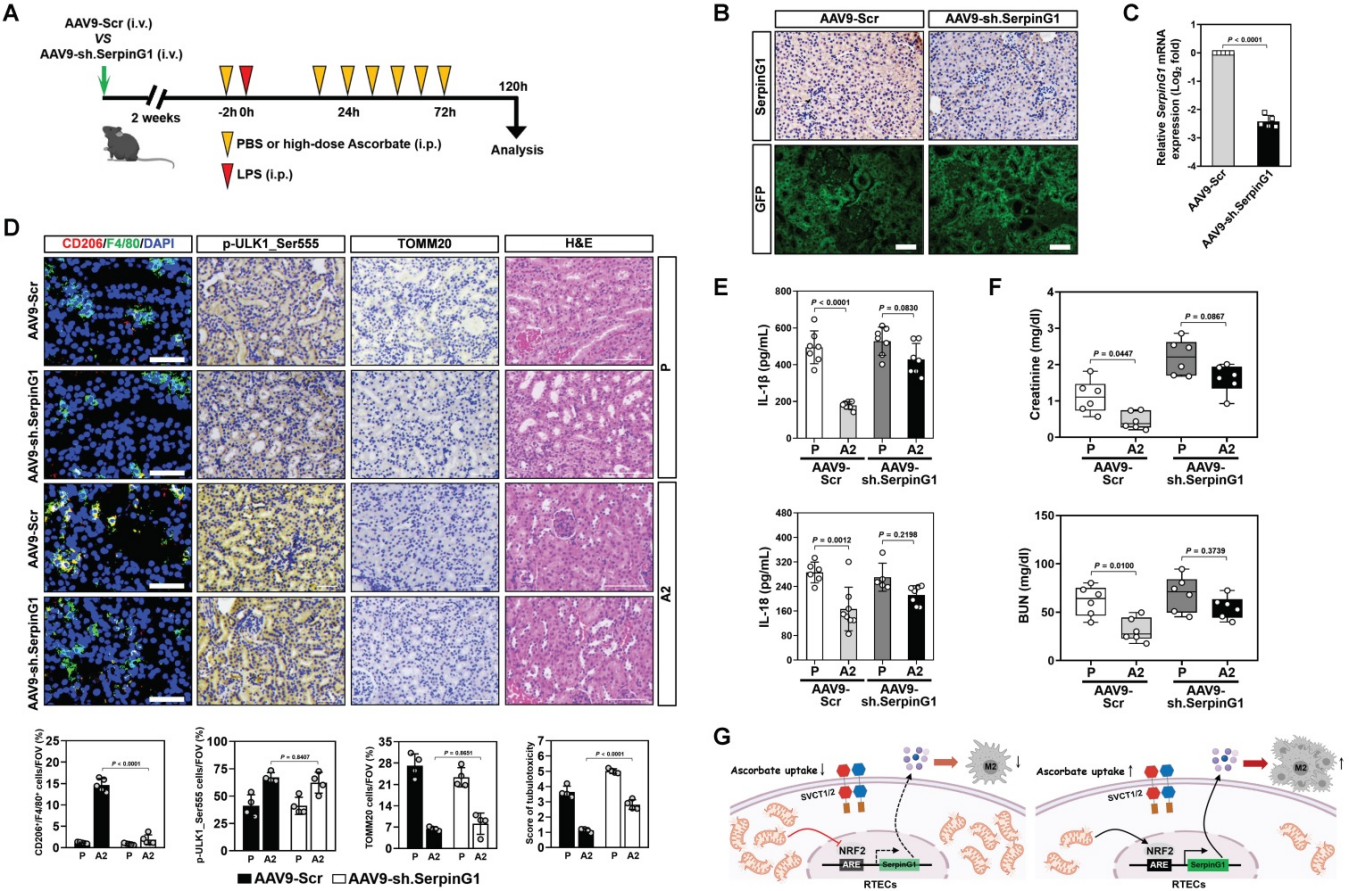
** SerpinG1 is required for the anti-septic AKI efficacy of high-dose ascorbate. (A)** Experimental scheme illustrating therapeutic regimen of high-dose ascorbate for LIE mice that were infected with AAV9-sh.SerpinG1 or the control vector AAV9-Scr at the indicated time points. **(B)** Representative images of SerpinG1 and GFP staining in renal sections from mice with AAV9-sh.SerpinG1 or AAV9-Scr infection. Scale bar: 100 μm. **(C)** RT-qPCR analysis comparing mRNA expression of *SerpinG1* in renal tissues from mice with AAV9-sh.SerpinG1 or AAV9-Scr infection (*n* = 5 per group). Data are expressed as mean ± s.d. Experiments were performed five times, each with quantitative RT-PCR in technical duplicate and real-time values were normalized to glyceraldehyde 3-phosphate dehydrogenase (GAPDH). Two-sided ANOVA with Bonferroni post hoc *t* test correction was used to calculate the *P* value. **(D)** Representative images (top panel) and quantification (bottom panel) of CD206^+^/F4/80^+^, p-ULK1_Ser555 and H&E staining in renal sections from LIE mice with AAV9-sh.SerpinG1 or AAV9-Scr infection in the presence of PBS (P) or high-dose ascorbate (A2) therapy (*n* = 4 per group). Data are expressed as mean ± s.d. Two-sided ANOVA with Bonferroni post hoc *t* test correction was used to calculate the *P* value. **(E)** ELISA measuring secretion of interleukin-1β (IL-1β) and interleukin-18 (IL-18) in kidney homogenate of LIE mice with AAV9-sh.SerpinG1 or AAV9-Scr infection in the presence of PBS (P) or high-dose ascorbate (A2) therapy (*n* ≥ 5 per group). Data are expressed as mean ± s.d. Two-sided ANOVA with Bonferroni post hoc *t* test correction was used to calculate the *P* value. **(F)** Serum creatinine (Scr) and blood urea nitrogen (BUN) levels in LIE mice with AAV9-sh.SerpinG1 or AAV9-Scr infection in the presence of PBS (P) or high-dose ascorbate (A2) therapy (*n* = 6 per group). Data are expressed as mean ± s.d. Two-sided ANOVA with Bonferroni post hoc *t* test correction was used to calculate the *P* value. **(G)** Schematic models of the role of tubular mitophagy-dependent SerpinG1 in the high-dose ascorbate-inducible M2 macrophages polarization mediated by NRF2 transactivation.

To interrogate the relevance of SerpinG1 to the anti-septic AKI efficacy of high-dose ascorbate, mice were injected with AAV9-Ksp-GFP-shSerpinG1 (AAV9-sh.SerpinG1) or the control vector AAV9-Ksp-GFP-shScramble (AAV9-Scr) (Figure 7A). SerpinG1 protein expression was greatly reduced in renal tissues from mice receiving AAV9-sh.SerpinG1 delivery, whose infection efficiency in renal tubular epithelial cells was equivalent to AAV9-Scr delivery (Figure 7B, C). The proportion of CD206^+^/F4/80^+^ macrophages in the AAV9-sh.SerpinG1-delivered LIE mice were lower than those in the AAV9-Scr-delivered LIE mice, and high-dose ascorbate lost the ability to increase CD206^+^/F4/80^+^ macrophages in the AAV9-sh.SerpinG1-delivered LIE mice as did in the AAV9-Scr-delivered LIE mice (Figure 7D). However, the proportion of p-ULK1_Ser555-positive cells in the AAV9-sh.SerpinG1-delivered LIE mice did not differ significantly from those in the AAV9-Scr-delivered LIE mice irrespective of high-dose ascorbate therapy. AAV9-sh.SerpinG1 delivery exacerbated tubulotoxicity of LIE mice and abrogated tubular protection by high-dose ascorbate therapy (Figure 7D). High-dose ascorbate appreciably decreased production of renal IL-1β and IL-18 in AAV9-Scr-delivered LIE mice, while there was no significant difference between the AAV9-sh.SerpinG1-delivered LIE mice with or without high-dose ascorbate therapy (Figure 7E). Upon LIE challenge, the levels of Scr and BUN were dropped in the AAV9-Scr-delivered mice following high-dose ascorbate therapy, whereas such reduction was not statistically significant in the AAV9-sh.SerpinG1-delivered mice (Figure 7F). These data demonstrate that tubular SerpinG1 mediates anti-inflammatory macrophage polarization to prevent septic AKI in response to high-dose ascorbate therapy.

## Discussion

Despite conventional therapy has achieved some clinical benefits in the past decades, prognosis of vast majority of patients with septic AKI is still unfavorable. Inflammatory invasion and stromal signals interdependently form a feedback loop by which vicious influx of leukocytes deteriorates tissue damage 33. It is therefore conspicuous that discerning the precise mechanisms responsible for crosstalk between tubular cells and inflammatory immune cells is critical for the development of novel therapeutic strategies. In this study, we underscore an underappreciated role of high-dose ascorbate in preventing septic AKI due to the regulatory interaction of tubular mitophagy with anti-inflammatory macrophage polarization. Elucidation of the genetic and molecular mechanisms underlying this event makes a combination strategy to overcome resistance of conventional agents possible. In proof-of-principle studies, we establish that the tubular mitophagy-dependent SerpinG1 secretion mediated by NRF2 transactivation contributes to the anti-inflammatory macrophage polarization and anti-septic AKI effects of high-dose ascorbate therapy (Figure 7G), suggesting that renal SerpinG1 might serve as a biomarker to predict responsiveness of high-dose ascorbate therapy. Our future work will aim at ascertaining alterations of other immunocyte subsets in response to high-dose ascorbate therapy and gaining insights into the clinical correlation of NRF2 with SerpinG1 in patients with septic AKI.

It is noteworthy that the anti-septic role of pharmacologic ascorbate is mainly mediated by sustaining activity of anti-oxidant enzymes in subcellular compartment 34. Multiple mechanisms are manifested in different studies, including vasopressin synthesis 35, superoxide sequestration 36, ferritin iron mobilization 37, folate metabolism 38 and, as we reported recently, mitophagy 17. So it remains inconclusive how ascorbate exerts its selective anti-septic AKI effects and it is difficult to define a convincing theory for explanation of these phenomena. Here, we unearth that high-dose ascorbate triggers tubular SerpinG1 secretion, which in turn facilitates polarization of the anti-inflammatory macrophages. We do not exclude other molecular mechanisms because ascorbate uptake might regulate various cellular events to direct function and activity of macrophages. The mechanistic data in our present study expand the repertoires reported previously, as SerpinG1 has been linked to suppression of inflammation, oxidative stress, cell apoptosis and necrosis 39-41. Understanding of the precise mechanism might also provide rationale for synergy of high-dose ascorbate together with other anti-septic AKI agents.

We demonstrate that the tubular mitophagy-dependent NRF2 transactivation participates in the high-dose ascorbate-inducible SerpinG1 secretion during septic AKI. This finding reinforces the concept that restoration of tubular mitophagy renders nuclear translocation and anti-oxidant activity of NRF2 42, 43. Reciprocally, NRF2 activation boosts mitophagy and injury repair in a PINK1/PARK2-dependent manner 44. NRF2 also contributes to the anti-septic AKI phenotypes followed by anti-oxidant supplementation 45, 46. Consistently, we demonstrate that tubular mitophagy stimulates SerpinG1 secretion in the setting of sustainable NRF2 transactivation. Our study provide complementary evidence that NRF2 transactivation blunts septic AKI and that tubular SerpinG1 secretion can be driven by endogenous anti-oxidative molecules in the presence of high-dose ascorbate.

One limitation of the current study is that the LIE models only allow us to study the impact of high-dose ascorbate on septic AKI elicited by pathogen-associated molecular patterns (PAMPs), but not by gram-positive and/or -negative bacteria. Ascorbate is found to be protective against vascular leakage in cecal ligation and puncture (CLP) mice 47, so it is possible that the high-dose ascorbate-polarized anti-inflammatory macrophages might be required for septic AKI prevention in that setting. Another limitation is that although our findings speculate that high-dose ascorbate stimulates SerpinG1 secretion via tubular mitophagy, the detailed mechanisms of how high-dose ascorbate protects clinical patients against septic AKI remain unclear. It would be of interest to conduct larger clinical trials to determine whether the anti-septic AKI phenotypes of high-dose ascorbate was dependent on tubular mitophagy and SerpinG1. Further studies also need to be done to explore benefit of combing high-dose ascorbate with conventional anti-septic AKI agents definitively.

In summary, we uncover that mitophagy/NRF2/SerpinG1 axis is fundamental for the macrophages-driven anti-septic AKI immunity and identify tubular SerpinG1 as a predictive marker for high-dose ascorbate efficacy. Our data also support the notion that intravenous high-dose ascorbate supplementation in clinical patients may augment efficacy of either the antibiotics-based treatment or renal replacement therapy and reduce mortality in hospital.

## Supplementary Material

Supplementary figures and tables.
