# Supplementary material for: Tubule-mitophagic secretion of SerpinG1 reprograms macrophages to instruct anti-septic acute kidney injury efficacy of high-dose ascorbate mediated by NRF2 transactivation
Source: Int J Biol Sci. 2022 Aug 8;18(13):5168–84. doi: 10.7150/ijbs.74430 (PMC9379417; doi:10.7150/ijbs.74430)
Supplement: Supplementary file 1 — Supplementary figures and tables. [file ijbsv18p5168s1.pdf]

Tubule-mitophagic secretion of SerpinG1 reprograms macrophages to instruct anti-septic acute kidney injury efficacy of high-dose ascorbate mediated by NRF2 transactivation

Yin Ni[1], Guo-Hua Wu[2], Juan-Juan Cai[3], Run Zhang[1], Yang Zheng[1], Jing-Quan Liu[1], Xiang-Hong Yang[1], Xue Yang[4], Ye Shen[5, 6], Jun-Mei Lai[5, 6], Xiang-Ming Ye[5, 6], Shi-Jing Mo[5, 6, 1*]

[1]*Emergency and Intensive Care Unit Center, Department of Intensive Care Unit, Zhejiang Provincial People's Hospital, Affiliated People's Hospital, Hangzhou Medical College, Hangzhou 310014, Zhejiang, P.R.China*

[2]*Zhejiang University School of Medicine, Zhejiang University, Hangzhou 310029, Zhejiang, P.R.China*

[3]*Department of Pathology, Zhejiang Provincial People's Hospital, Affiliated People's Hospital, Hangzhou Medical College, Hangzhou 310014, Zhejiang, P.R.China*

[4]*Clinical Research Institute, Zhejiang Provincial People's Hospital, Affiliated People's Hospital, Hangzhou Medical College, Hangzhou 310014, Zhejiang, P.R.China*

[5]*Center for Rehabilitation Medicine, Department of Intensive Rehabilitation Care Unit, Zhejiang Provincial People's Hospital, Affiliated People's Hospital, Hangzhou Medical College, Hangzhou 310014, Zhejiang, P.R.China*

[6]*Center for Rehabilitation Medicine, Rehabilitation & Sports Medicine Research Institute of Zhejiang Province, Department of Rehabilitation Medicine, Zhejiang Provincial People's Hospital, Affiliated People's Hospital, Hangzhou Medical College, Hangzhou 310014, Zhejiang, P.R.China*

[*]**Corresponding author:**
Shi-Jing Mo, MD, PhD
Department of Intensive Rehabilitation Care Unit & Rehabilitation Medicine
Department of Intensive Care Unit
Zhejiang Provincial People's Hospital
Affiliated People's Hospital, Hangzhou Medical College
Hangzhou, Zhejiang 310014
P.R. China
Email : moshijing@hmc.edu.cn
ORCID: https://orcid.org/0000-0002-1303-4766

Supplemental Figure S1-7 and Supplemental Table S1 and 2

Supplemental Figure S1. High-dose ascorbate by itself did not prime macrophages for phenotypic switch during septic AKI. (A and B) ELISA measuring interleukin-1β (IL-1β) and interleukin-18 (IL-18) production in kidney homogenate of LPS-induced endotoxemia (LIE) mice with PBS (P), low- or high-dose ascorbate (A1 or A2) therapy (n = 9 per group). Data are expressed as mean ± s.d. Two-sided ANOVA with Bonferroni post hoc t test correction was used to calculate the P value. Iso, isotype. **(C)** Fluorescence-activated cell sorting (FACS) analyses comparing F4/80$^+$ proportion in spleen tissues of mice with or without intravenous administration of clodronate liposome (Clod) treatment. **(D)** Experimental scheme illustrating adoptive transfer of splenic macrophages (Mac) from mice with either PBS (P) or high-dose ascorbate (A2) therapy into LIE mice in which macrophages were depleted by clodronate liposomes in the presence of PBS or high-dose ascorbate therapy. **(E-I)** Representative images and quantification of iNOS$^+$/F4/80$^+$, CD206$^+$/F4/80$^+$, H&E and p-ULK1_Ser555 staining in renal sections from clodronate liposomes-pretreated mice with P or A2 splenic macrophages transfer in the presence of PBS or high-dose ascorbate therapy upon LIE challenge. Data are expressed as mean ± s.d. Two-sided ANOVA with Bonferroni post hoc t test correction was used to calculate the P value ($n \geq 5$ per group). Scale bar: 50 μm and 100 μm. **(J and K)** ELISA assays measuring interleukin-1β (IL-1β) and interleukin-18 (IL-18) production in kidney homogenate of clodronate liposomes-pretreated mice with P or A2 splenic macrophages transfer in the presence of PBS or high-dose ascorbate therapy upon LIE challenge ($n \geq 5$ per group). Data are expressed as mean ± s.d. Two-sided ANOVA with Bonferroni post hoc t test correction was used to calculate the P value.

Supplemental Figure S2. Mitophagy inhibition or SVCT-1 and -2 knockout in RTECs abrogates the boosting effects of high-dose ascorbate on M2 macrophages. (A and B) Representative FACS histograms examining CD206$^+$ populations as well as ELISA comparing IL-10 and TNF productions in BMDMs incubated with conditioned medium (CM) from the scrambled shRNA (Scr)- or *Atg7* shRNA (sh.*Atg7*)-transfected and DMSO- or liensinine (Lien)-pretreated RTECs under LPS plus high-dose ascorbate-costimulated circumstances, respectively ($n \geq 3$ per group). Data are expressed as mean ± s.d. Two-sided ANOVA with Bonferroni post hoc t test correction was used to calculate the P value. **(C)** Western-blotting analyses determining ATG7 expression in RTECs transfected with scrambled shRNA (Scr) or *Atg7* shRNA (sh.*Atg7*). **(D)** Representative FACS histograms evaluating CD206$^+$ populations in BMDMs incubated with conditioned medium (CM) from the

SVCT1- and/or SVCT2-knockout (KO) RTECs under LPS plus high-dose ascorbate-costimulated circumstances, respectively (n = 3 per group). Data are expressed as mean ± s.d. Two-sided ANOVA with Bonferroni post hoc t test correction was used to calculate the P value. **(E)** Western-blotting analyses measuring SVCT-1 and -2 expression in RTECs where SVCT1- and/or SVCT2 were knocked out by CRISPR-Cas9 genome editing. **(F)** Western-blotting analyses assessing levels of iNOS and ARG1 protein expression in SVCT1- and/or SVCT2-knockout (KO) BMDMs stimulated with LPS (n = 3 per group).

Supplemental Figure S3. Tubular epithelium-specific ATG7 ablation impairs renal protection *in vivo* and anti-inflammatory macrophages polarization *in vitro* elicited by high-dose ascorbate. (A) Left panel: Representative gel images for the genotyping PCR to verify the genotype of $ATG7^{\Delta TE}$ mice (n = 3). Right panel: Representative immunofluorescence (IF) images of ATG7 in mouse primary RTECs. **(B)** Representative immunohistochemistry (IHC) images of p62 and ATG7 protein expression as well as transmission electron microscopy (TEM) pictures of mitophagosomes in $Atg7^{flox/flox}$ and $Atg7^{\Delta TE}$ mice. Scale bar: 100 μm and 1 μm. **(C and D)** Serum creatinine (Scr) and blood urea nitrogen (BUN) levels in $Atg7^{flox/flox}$ and $Atg7^{\Delta TE}$ mice receiving PBS (P) or high-dose ascorbate (A2) therapy upon LIE challenge ($n \geq 10$ per group). Data are expressed as mean ± s.d. Two-sided ANOVA with Bonferroni post hoc t test correction was used to calculate the P value. **(E)** Kaplan-Meier curves comparing survivals of $Atg7^{flox/flox}$ and $Atg7^{\Delta TE}$ mice receiving PBS (P) or high-dose ascorbate (A2) therapy upon LIE challenge ($n \geq 12$ mice per group). Log-rank t test was used to calculate the P value. **(F)** Representative images and quantification of H&E staining in renal sections from $Atg7^{flox/flox}$ and $Atg7^{\Delta TE}$ mice receiving PBS (P) or high-dose ascorbate (A2) therapy upon LIE challenge in the presence or absence of clodronate liposomes (Clod) pretreatment (n = 6 per group). Data are expressed as mean ± s.d. Two-sided ANOVA with Bonferroni post hoc t test correction was used to calculate the P value. Scale bar: 100 μm. **(G)** ELISA assessing secretion of interleukin-4 (IL-4) and interferon-γ (IFN-γ) in BMDMs incubated with conditioned medium (CM) from RTECs from $Atg7^{flox/flox}$ and $Atg7^{\Delta TE}$ mice under LPS plus high-dose ascorbate-costimulated circumstances, respectively (n = 4 per group). Data are expressed as mean ± s.d. Two-sided ANOVA with Bonferroni post hoc t test correction was used to calculate the P value. **(H)** Western-blotting analyses comparing ATG7 expression in $Atg7^{flox/flox}$ and $Atg7^{\Delta TE}$ RTECs.

Supplemental Figure S4. High-dose ascorbate favors tubular secretion of

SerpinG1 in a mitophagy-dependent fashion under inflammatory stress. (A) Quantification of DAB2, IGF1, SerpinG1, SerpinB2, TNC and VEGFC protein levels in cell lysates or conditioned medium (CM) from SVCT-1 and/or -2 knockout (KO) RTECs with LPS stimuli as in Figure 4B (n = 3 per group). Data are expressed as mean ± s.d. Two-sided ANOVA with Bonferroni post hoc t test correction was used to calculate the P value. **(B)** ELISA examining secretion of SerpinG1 in the LPS-stimulated RTECs with high-dose ascorbate exposure followed by withdrawal for the indicated times (n = 4 per group). Data are expressed as mean ± s.d. Two-sided ANOVA with Bonferroni post hoc t test correction was used to calculate the P value. **(C)** RT-qPCR analysis assessing mRNA expression of *SerpinG1* in the SVCT-1 and -2 shRNA-transfected HK-2 cells with LPS and/or high-dose ascorbate costimuli in the presence or absence of reconstituted SVCT-1 plus -2 expression (n = 7 per group). Data are expressed as mean ± s.d. Two-sided ANOVA with Bonferroni post hoc t test correction was used to calculate the P value. **(D)** Quantification of SerpinG1 protein levels in the SVCT-1 and -2 shRNA-transfected HK-2 cells with LPS and/or high-dose ascorbate costimuli in the presence or absence of reconstituted SVCT-1 plus -2 expression as in Figure 4D (n = 3 per group). Data are expressed as mean ± s.d. Two-sided ANOVA with Bonferroni post hoc t test correction was used to calculate the P value. **(E)** Western-blotting analyses determining abundance of SerpinG1 in renal tissues from $Atg7^{\mathrm{flox/flox}}$ and $Atg7^{\Delta\mathrm{TE}}$ mice subjected to LIE (n = 3 per group). **(F)** RT-qPCR analysis measuring mRNA expression of *SerpinG1* in $Atg7^{\mathrm{flox/flox}}$ and $Atg7^{\Delta\mathrm{TE}}$ RTECs with LPS stimuli (n = 5 per group). Data are expressed as mean ± s.d. Experiments were performed five times, each with quantitative RT-PCR in technical duplicate and real-time values were normalized to glyceraldehyde 3-phosphate dehydrogenase (GAPDH). Two-sided ANOVA with Bonferroni post hoc t test correction was used to calculate the P value. **(G)** ELISA examining secretion of SerpinG1 in $Atg7^{\mathrm{flox/flox}}$ and $Atg7^{\Delta\mathrm{TE}}$ RTECs with LPS stimuli (n = 4 per group). Data are expressed as mean ± s.d. Two-sided ANOVA with Bonferroni post hoc t test correction was used to calculate the P value. **(H)** Left panel: ELISA detecting secretion of SerpinG1 in the LPS-stimulated RTECs transfected with siRNA targeting PINK1, PARK2 or NIX, respectively (n = 4 per group). Right panel: Western-blotting analyses detecting levels of PINK1, PARK2 or NIX in RTECs transfected with siRNA targeting PINK1, PARK2 or NIX, respectively. Un, untransfected. M, mock. **(I)** ELISA comparing secretion of SerpinG1 in the LPS-stimulated HK-2 cells with or without CCCP (10 μmol/L) treatment (n = 4 per group). Data are expressed as mean ± s.d. Two-sided ANOVA with Bonferroni post hoc t test correction was used to calculate the P value. **(J)** TOMM20 staining, cytosolic mitochondrial COX-1

(mt-COX-1) release and mitochondrial reactive oxygen species (mt-ROS) production of the LPS-stimulated RTECs with recombinant SerpinG1 (rSerpinG1, 100 μg/mL) or high-dose ascorbate treatment (*n* = 3 per group). Data are expressed as mean ± s.d. Two-sided ANOVA with Bonferroni post hoc *t* test correction was used to calculate the *P* value. **(K)** TOMM20 staining, cytosolic mitochondrial COX-1 (mt-COX-1) release and mitochondrial reactive oxygen species (mt-ROS) production of the LPS-stimulated and LPS plus high-dose ascorbate-costimulated RTECs with SerpinG1 siRNA transfection (*n* = 4 per group). Data are expressed as mean ± s.d. Two-sided ANOVA with Bonferroni post hoc *t* test correction was used to calculate the *P* value. si.Ctrl: control siRNA. **(L)** ELISA evaluating secretion of SerpinG1 in the LPS and/or high-dose ascorbate-costimulated RTECs with liensinine (Lien) treatment in the presence of scrambled shRNA (Scr) or *Atg7* shRNA (sh.*Atg7*) transfection (*n* = 4 per group). Data are expressed as mean ± s.d. Two-sided ANOVA with Bonferroni post hoc *t* test correction was used to calculate the *P* value.

Supplemental Figure S5. NRF2 transactivation contributes to the high-ascorbate-inducible SerpinG1 secretion mediated by tubular mitophagy. (A) ELISA determining secretion of SerpinG1 in the LPS plus high-dose ascorbate-costimulated RTECs with alkaloid trigonelline (Trig.) treatment for the indicated times (*n* = 6 per group). Data are expressed as mean ± s.d. Two-sided ANOVA with Bonferroni post hoc *t* test correction was used to calculate the *P* value. **(B)** ELISA comparing secretion of SerpinG1 in the LPS-stimulated RTECs with HA-tagged NRF2 expression (*n* = 4 per group). Data are expressed as mean ± s.d. Two-sided ANOVA with Bonferroni post hoc *t* test correction was used to calculate the *P* value. Un: untransfected; EV: empty vector. **(C)** Western-blotting analyses detecting levels of SerpinG1 protein in RTECs with HA-tagged NRF2 expression. **(D)** Left panel: ELISA examining secretion of SerpinG1 in the LPS plus high-dose ascorbate-costimulated RTECs transfected with ATG7 shRNA (sh.*Atg7*) in the presence or absence of NRF2 depletion (*n* = 4 per group). Data are expressed as mean ± s.d. Two-sided ANOVA with Bonferroni post hoc *t* test correction was used to calculate the *P* value. Middle and right panel: western-blotting analyses comparing levels of NRF2 and ATG7 protein in RTECs transfected with NRF2 shRNA (sh.NRF2) and ATG7 shRNA (sh.*Atg7*), respectively.

Supplemental Figure S6. Tubular SerpinG1 perpetuates the anti-inflammatory macrophages and thereby prevents septic AKI. (A) Western-blotting analyses examining abundance of SerpinG1 protein in RTECs transfected with vector

expressing GFP or GFP-tagged SerpinG1. CB: coomassie blue. **(B and C)** Representative contour plots and quantification of FACS assessing $CD206^+/CD86^-$ populations in the LPS-stimulated BMDMs with the indicated conditioned medium (CM) treatment in the presence of anti-SerpinG1 Ab incubation (*n* = 3 per group). Data are expressed as mean ± s.d. Two-sided ANOVA with Bonferroni post hoc *t* test correction was used to calculate the *P* value. **(D)** Western-blotting analyses (left panel) and quantification (right panel) testing abundance of iNOS and ARG1 protein in the LPS-stimulated BMDMs with the indicated conditioned medium (CM) treatment in the presence of anti-SerpinG1 Ab incubation (*n* = 3 per group). Data are expressed as mean ± s.d. Two-sided ANOVA with Bonferroni post hoc *t* test correction was used to calculate the *P* value. **(E)** ELISA evaluating secretion of interleukin-1β (IL-1β), interleukin-18 (IL-18), C-C motif chemokine ligand 3 (CCL3) and interferon-γ (IFN-γ) in the LPS-stimulated BMDMs with the indicated conditioned medium (CM) treatment in the presence of anti-SerpinG1 Ab incubation (*n* = 4 per group). Data are expressed as mean ± s.d. Two-sided ANOVA with Bonferroni post hoc *t* test correction was used to calculate the *P* value. **(F)** Western-blotting analyses comparing levels of SerpinG1 protein in RTECs transfected with siRNA targeting control (si.Ctrl) or SerpinG1 (si.SerpinG1). **(G)** Representative FACS histograms and quantification evaluating $CD206^+$ populations in the LPS-stimulated or LPS plus high-dose ascorbate-costimulated BMDMs cocultured with RTECs transfected with siRNA targeting control (si.Ctrl) or SerpinG1 (si.SerpinG1) (*n* = 3 per group). Data are expressed as mean ± s.d. Two-sided ANOVA with Bonferroni post hoc *t* test correction was used to calculate the *P* value. **(H)** Representative images (left panel) and quantification (right panel) of H&E staining in renal sections from LPS-induced endotoxemia (LIE) mice receiving rSerpinG1 administration in the presence or absence of clodronate liposomes pretreatment (*n* = 5 per group). Data are expressed as mean ± s.d. Two-sided ANOVA with Bonferroni post hoc *t* test correction was used to calculate the *P* value. Scale bar: 100 μm. **(I)** Kaplan-Meier curves assessing survival of LPS-induced endotoxemia (LIE) mice receiving rSerpinG1 administration in the presence or absence of clodronate liposomes pretreatment (*n* ≥ 12 mice per group). Log-rank t test was used to calculate the *P* value.

Supplemental Figure S7. SerpinG1 reverses depolarization of anti-inflammatory macrophages and septic AKI exacerbation triggered by tubular epithelium-specific ablation of ATG7. (A) Representative images and quantification of $CD206^+/F4/80^+$, ATG7 and H&E staining in renal sections from *Atg7*$^{flox/flox}$ and *Atg7*$^{\Delta TE}$ mice with or without receiving rSerpinG1 (800 μg) administration upon LIE

challenge ($n \geq 3$ per group). Data are expressed as mean ± s.d. Two-sided ANOVA with Bonferroni post hoc t test correction was used to calculate the P value. Scale bar: 50 μm and 100 μm. **(B)** ELISA measuring secretion of interleukin-1β (IL-1β) in kidney homogenate of *Atg7*$^{flox/flox}$ and *Atg7*$^{\Delta TE}$ mice with or without receiving rSerpinG1 administration upon LIE challenge ($n = 4$ per group). Data are expressed as mean ± s.d. Two-sided ANOVA with Bonferroni post hoc t test correction was used to calculate the P value.

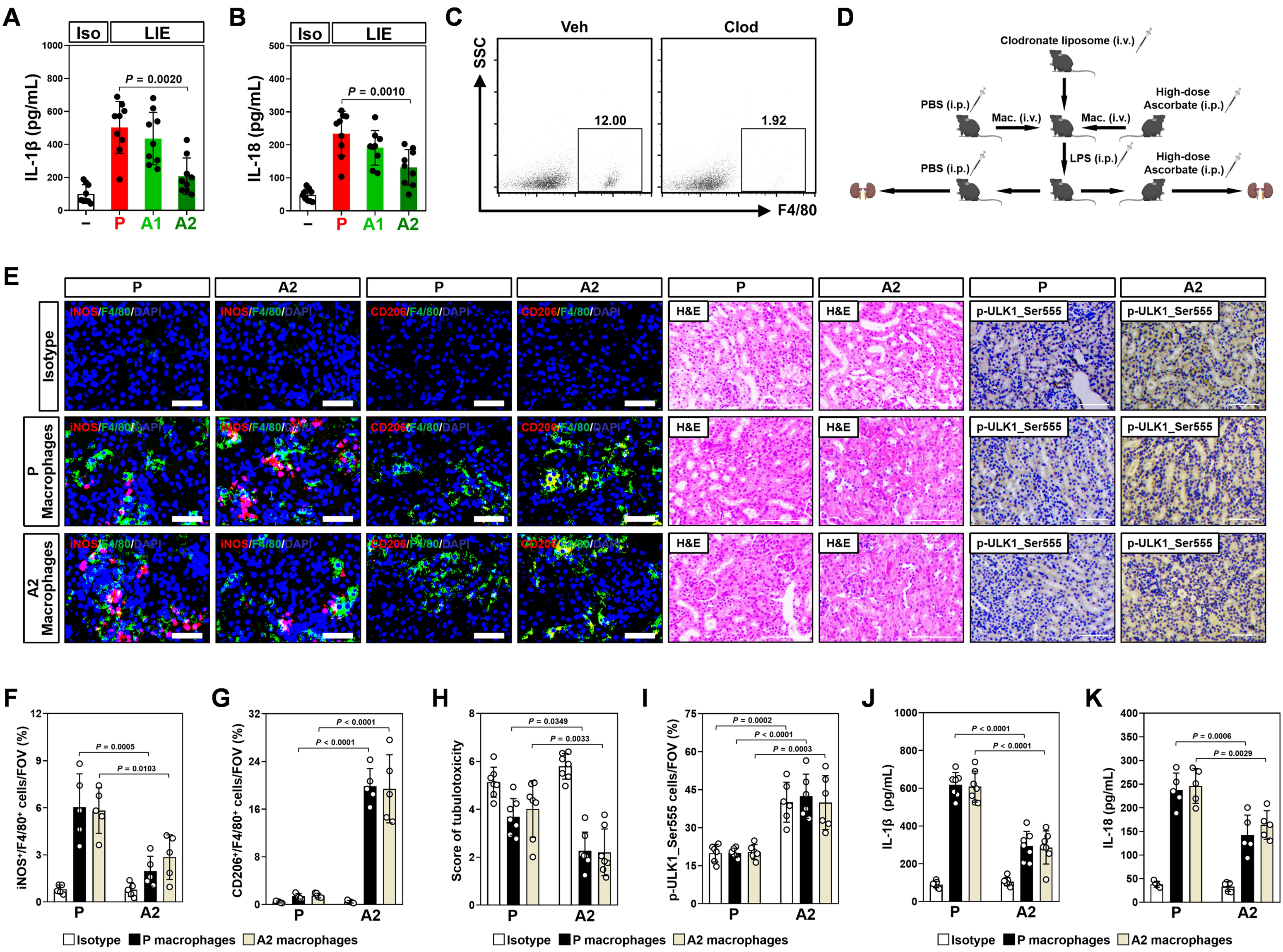

A
Iso
LIE
IL-1β (pg/mL)
P = 0.0020
– P A1 A2
B
IL-18 (pg/mL)
P = 0.0010
C
Veh
Clod
SSC
F4/80
12.00
1.92
D
Clodronate liposome (i.v.)
PBS (i.p.)
Mac. (i.v.)
High-dose Ascorbate (i.p.)
LPS (i.p.)
E
Isotype
P Macrophages
A2 Macrophages
iNOS/F4/80/DAPI
CD206/F4/80/DAPI
H&E
p-ULK1_Ser555
F
iNOS+/F4/80+ cells/FOV (%)
P = 0.0005
P = 0.0103
G
CD206+/F4/80+ cells/FOV (%)
P < 0.0001
H
Score of tubulotoxicity
P = 0.0349
P = 0.0033
I
p-ULK1_Ser555 cells/FOV (%)
P = 0.0002
P < 0.0001
P = 0.0003
J
IL-1β (pg/mL)
K
IL-18 (pg/mL)
P = 0.0006
P = 0.0029
Isotype
P macrophages
A2 macrophages

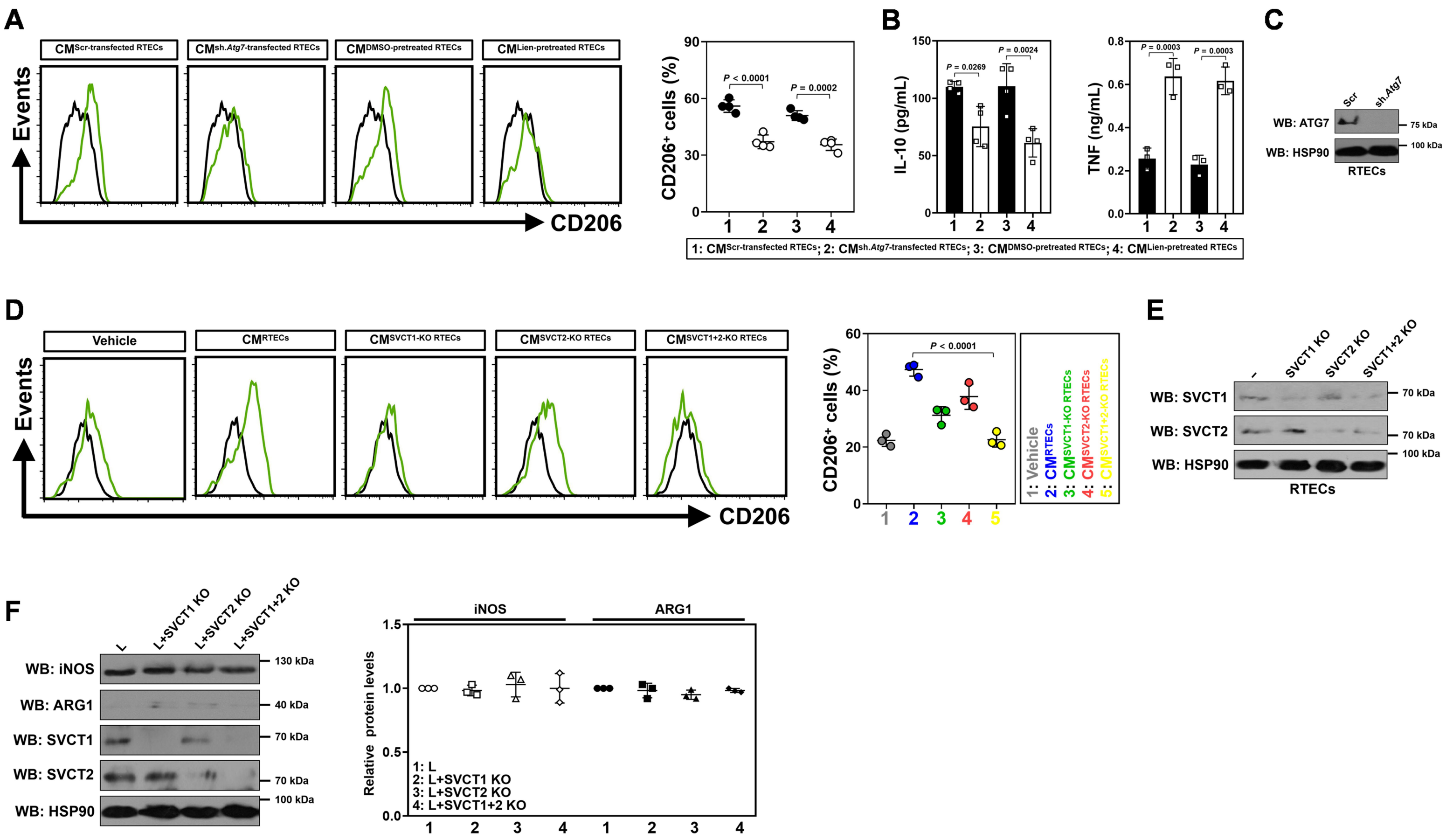

A
CM^Scr-transfected RTECs
CM^sh.Atg7-transfected RTECs
CM^DMSO-pretreated RTECs
CM^Lien-pretreated RTECs
Events
CD206
CD206+ cells (%)
P < 0.0001
P = 0.0002
1: CM^Scr-transfected RTECs; 2: CM^sh.Atg7-transfected RTECs; 3: CM^DMSO-pretreated RTECs; 4: CM^Lien-pretreated RTECs
B
IL-10 (pg/mL)
P = 0.0269
P = 0.0024
TNF (ng/mL)
P = 0.0003
P = 0.0003
C
Scr
sh.Atg7
WB: ATG7
WB: HSP90
75 kDa
100 kDa
RTECs
D
Vehicle
CM^RTECs
CM^SVCT1-KO RTECs
CM^SVCT2-KO RTECs
CM^SVCT1+2-KO RTECs
Events
CD206
CD206+ cells (%)
P < 0.0001
1: Vehicle
2: CM^RTECs
3: CM^SVCT1-KO RTECs
4: CM^SVCT2-KO RTECs
5: CM^SVCT1+2-KO RTECs
E
–
SVCT1 KO
SVCT2 KO
SVCT1+2 KO
WB: SVCT1
WB: SVCT2
WB: HSP90
70 kDa
70 kDa
100 kDa
RTECs
F
L
L+SVCT1 KO
L+SVCT2 KO
L+SVCT1+2 KO
WB: iNOS
WB: ARG1
WB: SVCT1
WB: SVCT2
WB: HSP90
130 kDa
40 kDa
70 kDa
70 kDa
100 kDa
iNOS
ARG1
Relative protein levels
1: L
2: L+SVCT1 KO
3: L+SVCT2 KO
4: L+SVCT1+2 KO

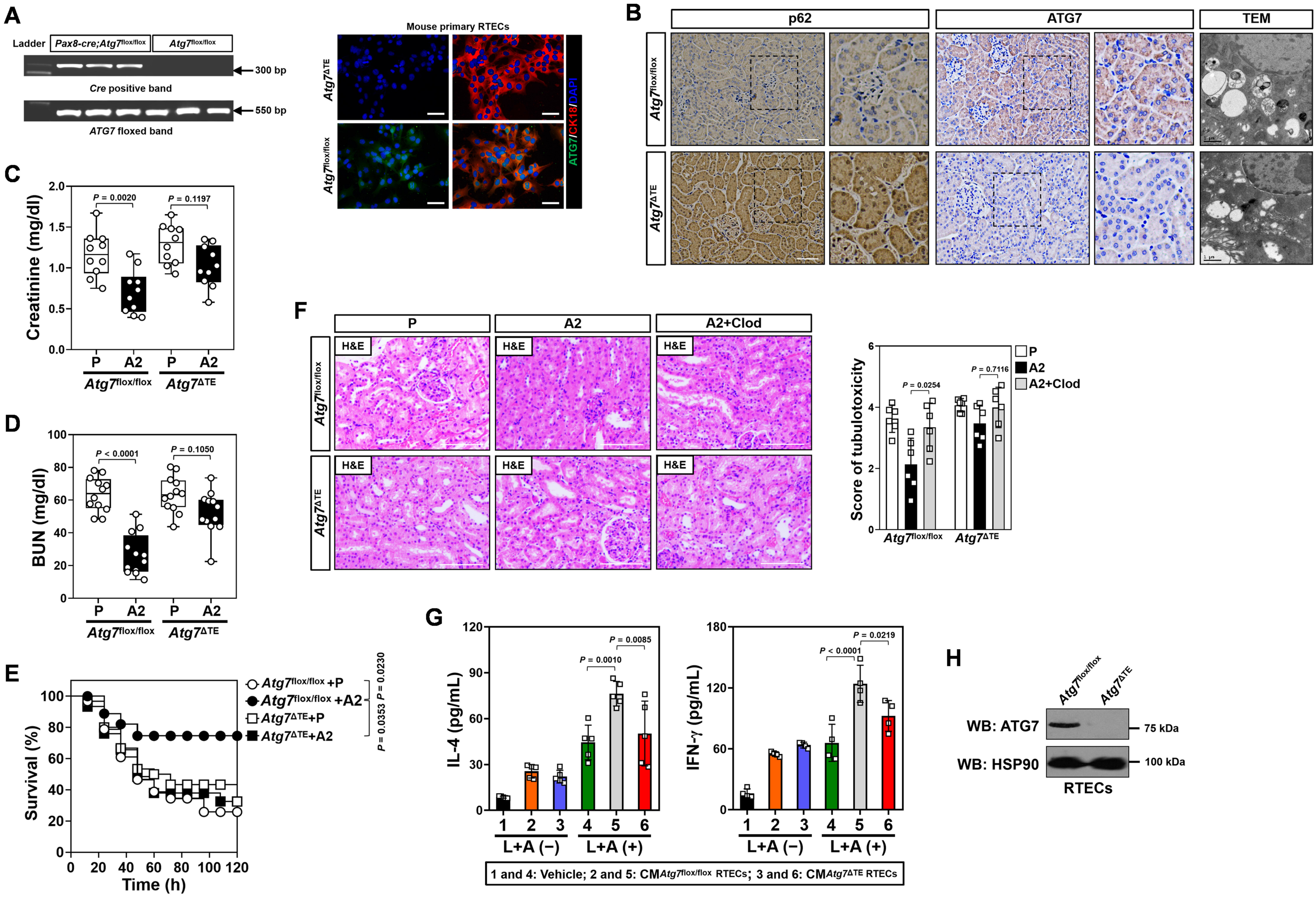

A
Ladder
Pax8-cre;Atg7flox/flox
Atg7flox/flox
300 bp
Cre positive band
550 bp
ATG7 floxed band
Mouse primary RTECs
Atg7ΔTE
Atg7flox/flox
ATG7/CK18/DAPI
B
p62
ATG7
TEM
C
Creatinine (mg/dl)
P = 0.0020
P = 0.1197
P A2 P A2
D
BUN (mg/dl)
P < 0.0001
P = 0.1050
E
Survival (%)
Time (h)
Atg7flox/flox+P
Atg7flox/flox+A2
Atg7ΔTE+P
Atg7ΔTE+A2
P = 0.0353
P = 0.0230
F
P
A2
A2+Clod
H&E
Score of tubulotoxicity
P = 0.0254
P = 0.7116
G
IL-4 (pg/mL)
P = 0.0010
P = 0.0085
IFN-γ (pg/mL)
P < 0.0001
P = 0.0219
L+A (−)
L+A (+)
1 and 4: Vehicle; 2 and 5: CMAtg7flox/flox RTECs; 3 and 6: CMAtg7ΔTE RTECs
H
WB: ATG7
WB: HSP90
75 kDa
100 kDa
RTECs

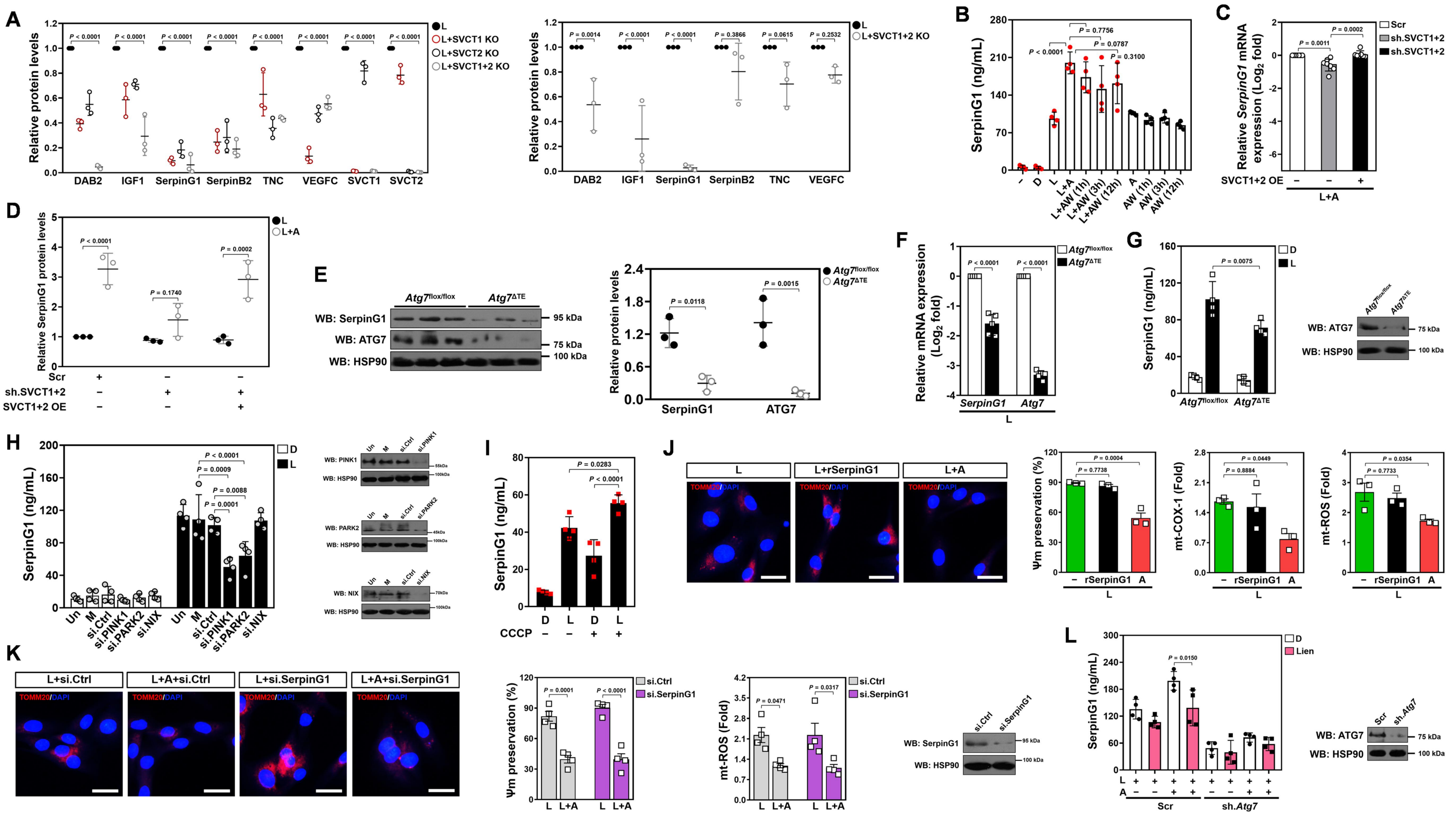
A
Relative protein levels
L
L+SVCT1 KO
L+SVCT2 KO
L+SVCT1+2 KO
DAB2
IGF1
SerpinG1
SerpinB2
TNC
VEGFC
SVCT1
SVCT2
L+SVCT1+2 KO
B
SerpinG1 (ng/mL)
C
Relative SerpinG1 mRNA expression (Log2 fold)
Scr
sh.SVCT1+2
SVCT1+2 OE
L+A
D
Relative SerpinG1 protein levels
L+A
E
WB: SerpinG1
WB: ATG7
WB: HSP90
95 kDa
75 kDa
100 kDa
ATG7
F
Relative mRNA expression (Log2 fold)
G
WB: ATG7
WB: HSP90
H
WB: PINK1
WB: PARK2
WB: NIX
I
CCCP
J
L+rSerpinG1
TOMM20/DAPI
Ψm preservation (%)
mt-COX-1 (Fold)
mt-ROS (Fold)
rSerpinG1
K
L+si.Ctrl
L+A+si.Ctrl
L+si.SerpinG1
L+A+si.SerpinG1
si.Ctrl
si.SerpinG1
WB: SerpinG1
WB: HSP90
L
Lien
sh.Atg7

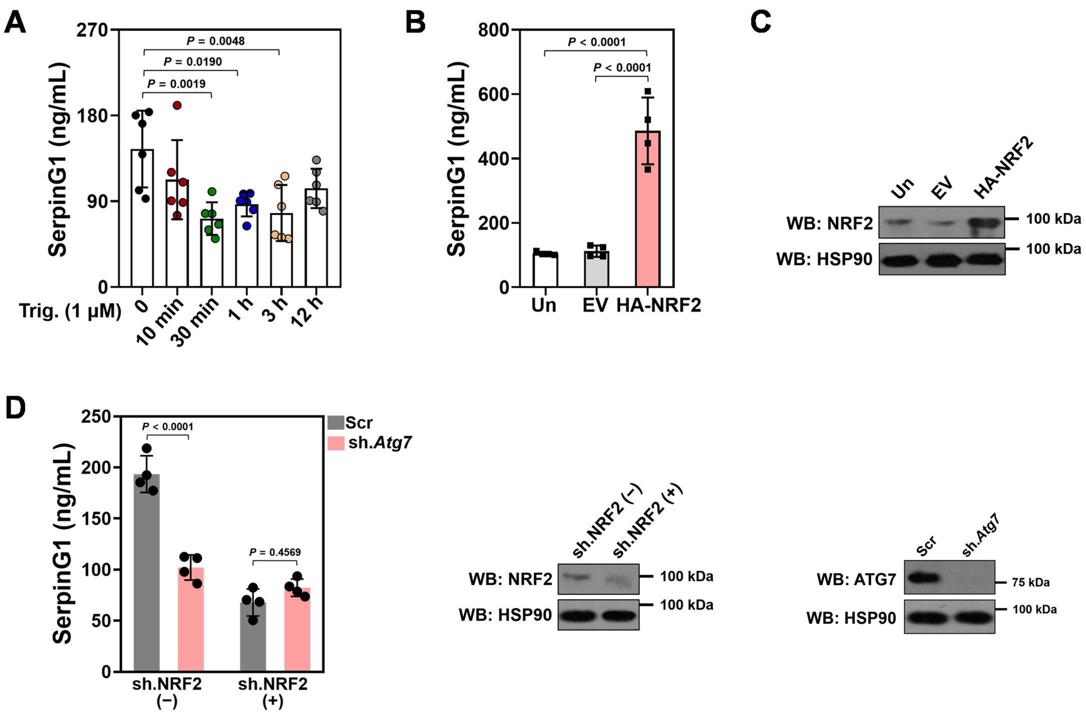

A
SerpinG1 (ng/mL)
270
180
90
0
P = 0.0048
P = 0.0190
P = 0.0019
Trig. (1 μM)
0
10 min
30 min
1 h
3 h
12 h
B
SerpinG1 (ng/mL)
800
600
400
200
0
P < 0.0001
P < 0.0001
Un
EV
HA-NRF2
C
Un
EV
HA-NRF2
WB: NRF2
WB: HSP90
100 kDa
100 kDa
D
SerpinG1 (ng/mL)
250
200
150
100
50
0
P < 0.0001
P = 0.4569
Scr
sh.*Atg7*
sh.NRF2 (−)
sh.NRF2 (+)
sh.NRF2 (−)
sh.NRF2 (+)
WB: NRF2
WB: HSP90
100 kDa
100 kDa
Scr
sh.*Atg7*
WB: ATG7
WB: HSP90
75 kDa
100 kDa

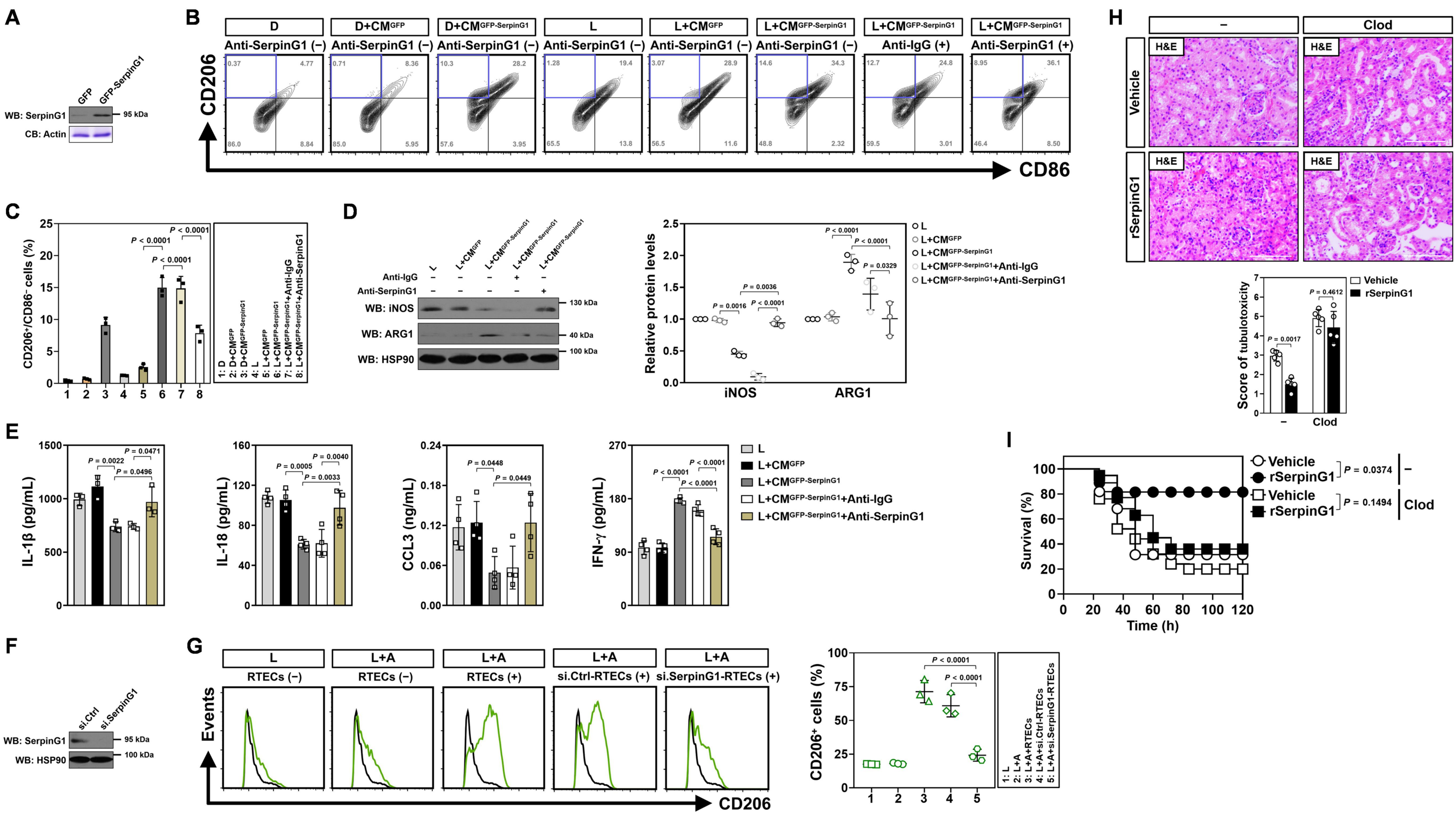
A
WB: SerpinG1
CB: Actin
GFP
GFP-SerpinG1
95 kDa
B
D
D+CMGFP
D+CMGFP-SerpinG1
L
L+CMGFP
L+CMGFP-SerpinG1
Anti-SerpinG1 (−)
Anti-IgG (+)
Anti-SerpinG1 (+)
CD206
CD86
C
CD206+/CD86− cells (%)
1: D
2: D+CMGFP
3: D+CMGFP-SerpinG1
4: L
5: L+CMGFP
6: L+CMGFP-SerpinG1
7: L+CMGFP-SerpinG1+Anti-IgG
8: L+CMGFP-SerpinG1+Anti-SerpinG1
D
WB: iNOS
WB: ARG1
WB: HSP90
Anti-IgG
Anti-SerpinG1
130 kDa
40 kDa
100 kDa
Relative protein levels
iNOS
ARG1
E
IL-1β (pg/mL)
IL-18 (pg/mL)
CCL3 (ng/mL)
IFN-γ (pg/mL)
L
L+CMGFP
L+CMGFP-SerpinG1
L+CMGFP-SerpinG1+Anti-IgG
L+CMGFP-SerpinG1+Anti-SerpinG1
F
WB: SerpinG1
WB: HSP90
si.Ctrl
si.SerpinG1
95 kDa
100 kDa
G
L
L+A
RTECs (−)
RTECs (+)
si.Ctrl-RTECs (+)
si.SerpinG1-RTECs (+)
Events
CD206
CD206+ cells (%)
1: L
2: L+A
3: L+A+RTECs
4: L+A+si.Ctrl-RTECs
5: L+A+si.SerpinG1-RTECs
H
–
Clod
Vehicle
rSerpinG1
H&E
Score of tubulotoxicity
P = 0.0017
P = 0.4612
I
Survival (%)
Time (h)
Vehicle
rSerpinG1
P = 0.0374
P = 0.1494
Clod

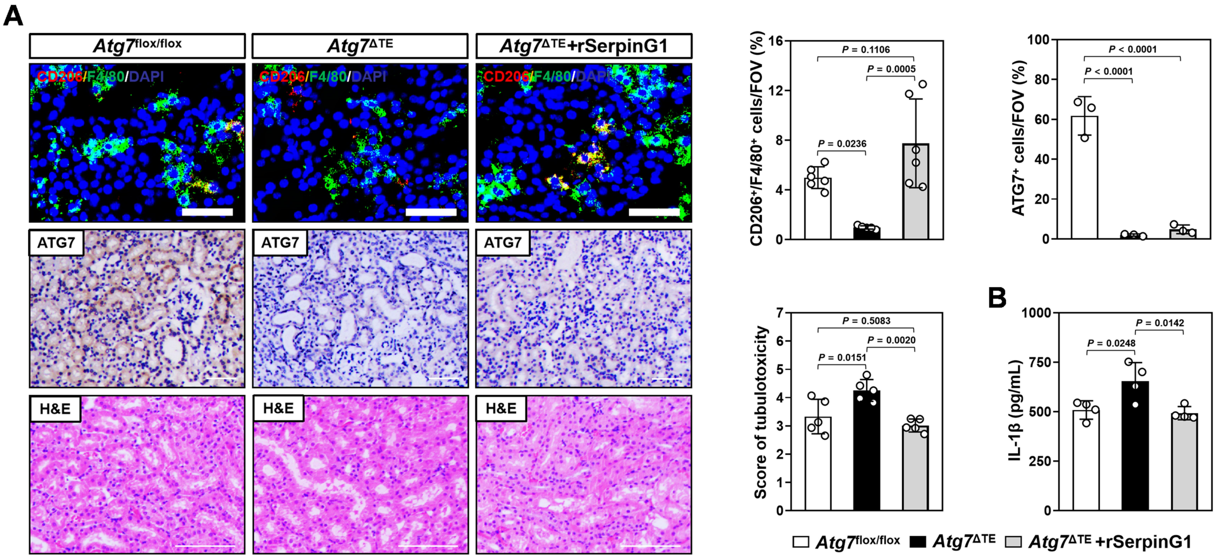

A
Atg7flox/flox
Atg7ΔTE
Atg7ΔTE+rSerpinG1
CD206/F4/80/DAPI
ATG7
H&E
CD206+/F4/80+ cells/FOV (%)
P = 0.1106
P = 0.0005
P = 0.0236
ATG7+ cells/FOV (%)
P < 0.0001
P < 0.0001
Score of tubulotoxicity
P = 0.5083
P = 0.0020
P = 0.0151
B
IL-1β (pg/mL)
P = 0.0142
P = 0.0248
Atg7flox/flox
Atg7ΔTE
Atg7ΔTE +rSerpinG1

Supplemental Table S1. A list of the secreted proteins significantly downregulated in SVCT1 and -2 KO RTECs relative to parental RTECs under LPS stimuli (Fold change ≤ −2)

Gene symbol	Description	Fold change	ANOVA *p* value	Chromosome
CTSB	Cathepsin B	-43.54	0.000455	Chr 14
LOXL4	Lysyl oxidase like 4	-40.58	0.000279	Chr 19
SERPING1	Serpin family G member 1	-36.64	0.009317	Chr 2
SERPINB2	Serpin family B member 2	-28.83	0.002078	Chr 1
SLURP1	Secreted LY6/PLAUR domain containing 1	-25.02	0.000542	Chr 15
FSHB	Follitropin subunit beta	-19.35	0.000375	Chr 2
COL12A1	Collagen alpha-1(XII) chain	-12.37	0.015754	Chr 9
DAB2	DAB adaptor protein 2	-12.06	0.009912	Chr 15
GALP	Galanin like peptide	-9.79	0.001775	Chr 7
IGF1	Insulin like growth factor 1	-7.31	0.014601	Chr 10
CCL2	C-C motif chemokine ligand 2	-5.53	0.022789	Chr 11
AMTN	Amelotin	-5.26	0.015077	Chr5
SFRP5	Secreted frizzled related protein 5	-5.12	0.000625	Chr 19
FAM20A	Pseudokinase FAM20A	-4.83	0.005566	Chr 11
TNC	Tenascin C	-4.12	0.007348	Chr 4
CXCL12	C-X-C motif chemokine 12	-4.06	0.047149	Chr 6
VEGFC	Vascular endothelial growth factor C	-3.07	0.003430	Chr 8
LGALS3BP	Galectin 3 binding protein	-2.93	0.024956	Chr 11

Supplemental Table S2. Primers used in real-time quantitative PCR (RT-qPCR)

**Gene**	**Accession number**	**Sense, 5′→3′**	**Antisense, 5′→3′**
DAB2-m	NM_023118	CCTTCATTGCTCGTGATGTGA	CCCCAAACAAATCCATCTGGTC
IGF1-m	NM_001111274	CACATCATGTCGTCTTCACACC	GGAAGCAACACTCATCCACAATG
SerpinG1-h	NM_001032295	CTGGCTGGGGATAGAGCCT	GAGATAACTGTTGTTGCGACCT
SerpinG1-m	NM_009776	GAGGCTCAAGCGAAAAGCAGA	ACTCGTTGGCTACTTTACCCA
CTSB-m	NM_007798	CAGGCTGGACGCAACTTCTAC	TCACCGAACGCAACCCTTC
LOXL4-m	NM_001164311	GCCAACGGACAGACCAGAG	CCAGGTCAAGGCTGACTCAAA
SLURP1-m	NM_020519	AGCCCACGGCCATTAACTC	CCAATGCCATCAGGGTCGG
FSHB-m	NM_008045	CCATAGCTGTGAATTGACCAACA	AGATCCCTAGTGTAGCAGTAGC
COL12A1-m	NM_007730	AGGCAGAAGTTGACCCACCT	CAGTGGTACTAGCTGCAAGGG
GALP-m	NM_178028	ATGGCCTGCTCCGTACATCT	ACCAGCACTATTGAGGGTCCA
CCL2-m	NM_011333	TAAAAACCTGGATCGGAACCAAA	GCATTAGCTTCAGATTTACGGGT
AMTN-m	NM_027793	ATCAGCCCAGTCATTACCAAAG	AGGTCTGACCCCAGAGTGAG
SFRP5-m	NM_018780	GAGATCAAGATAGACAACGGGGA	TTGCGCTTTAAGGGGCCTG
FAM20A-m	NM_153782	CTCCGCGTCTCTACACTCAG	GCCACCTTCCTCCGGTAATA
CXCL12-m	NM_001012477	TGCATCAGTGACGGTAAACCA	CACAGTTTGGAGTGTTGAGGAT
LGALS3BP-m	NM_011150	TGGTTCCAGGGACTCAAGGTA	CCACCGGCCTCTGTAGAAGA
SerpinB2-m	NM_001174170	ATTGGCAGTTATGGTATCACCAC	GGTGTGTTGATTGTTGAGCTGA
TNC-m	NM_011607	TTTGCCCTCACTCCCGAAG	AGGGTCATGTTTAGCCCACTC
VEGFC-m	NM_009506	GTGAGGTGTGTATAGATGTGGGG	ACGTCTTGCTGAGGTAACCTG
ATG7-m	NM_001253717	TCTGGGAAGCCATAAAGTCAGG	GCGAAGGTCAGGAGCAGAA
mtCOX-1-m	NC_005089	GCCCCAGATATAGCATTCCC	GTTCATCCTGTTCCTGCTCC
GAPDH-h	NM_001256799	GGAGCGAGATCCCTCCAAAAT	GGCTGTTGTCATACTTCTCATGG
GAPDH-m	NM_008084	AGGTCGGTGTGAACGGATTTG	GGGGTCGTTGATGGCAACA
